# Plant secondary metabolites induced electron flux in microbial fuel cell: investigation from laboratory-to-field scale

**DOI:** 10.1038/s41598-020-74092-y

**Published:** 2020-10-14

**Authors:** Dibyojyoty Nath, M. M. Ghangrekar

**Affiliations:** 1 School of Environmental Science and Engineering, Indian Institute of Technology Kharagpur, Kharagpur, 721302 India; 2 Department of Civil Engineering, Indian Institute of Technology Kharagpur, Kharagpur, 721302 India

**Keywords:** Biochemistry, Biotechnology, Energy science and technology

## Abstract

Wastewater treatment coupled with electricity recovery in microbial fuel cell (MFC) prefer mixed anaerobic sludge as inoculum in anodic chamber than pure stain of electroactive bacteria (EAB), due to robustness and syntrophic association. Genetic modification is difficult to adopt for mixed sludge microbes for enhancing power production of MFC. Hence, we demonstrated use of eco-friendly plant secondary metabolites (PSM) with sub-lethal concentrations to enhance the rate of extracellular electron transfer between EAB and anode and validated it in both bench-scale as well as pilot-scale MFCs. The PSMs contain tannin, saponin and essential oils, which are having electron shuttling properties and their addition to microbes can cause alteration in cell morphology, electroactive behaviour and shifting in microbial population dynamics depending upon concentrations and types of PSM used. Improvement of 2.1-times and 3.8-times in power densities was observed in two different MFCs inoculated with *Eucalyptus*-extract pre-treated mixed anaerobic sludge and pure culture of *Pseudomonas aeruginosa*, respectively, as compared to respective control MFCs operated without adding *Eucalyptus*-extract to inoculum. When *Eucalyptus*-extract-dose was spiked to anodic chamber (125 l) of pilot-scale MFC, treating septage, the current production was dramatically improved. Thus, PSM-dosing to inoculum holds exciting promise for increasing electricity production of field-scale MFCs.

## Introduction

Microbial fuel cell (MFC) generate electricity when electroactive microorganisms (EAM) respire at anode by extracellular electron transfer (EET) coupled with the oxidation of organic carbon^1^. These EAM have been identified across all three domains of life (Bacteria, Archaea and Eukaryotes) and carry out EET via three primary pathways, i.e. (1) *c*-type cytochromes (cyt *c*), (2) type IV pili/nanowire (membrane appendages or prosthecae) and (3) mediators^1^. The MFCs have broad applications including self-powered robots, to power the low-power devices such as illuminating light emitting diode bulbs, charging mobile phone and lithium ion batteries coupled with wastewater treatment^2,3^. Syntrophic interaction among mixed consortia results in higher power densities in MFCs than the most efficient pure culture electro-active bacteria (EAB)^1,4,5^. However, the genetic basis for syntrophic association is not well-understood and further improvement in the EET among mixed inoculum is not an easy task through genetic engineering approach^6^.

To improve electricity production in MFC having mixed anaerobic sludge microbes as inoculum, previously different physico-chemical and biological methods, e.g. high temperature (100 ˚C) or pH (in acidic range), addition of metabolites from algae and fungus (antibiotics) and 2-bromoethanosulphonate (BES), etc., were adopted to inhibit methanogenesis and increase in power density (PD) of MFC was reported^7–12^. However, inhibition of methanognesis is an indirect approach to boost electrogenesis in MFC; since, the correlation between the higher PD and response of EAM, for example whether EET is increased among EAMs, is unknown. Similarly, cisplatin was applied on *Shewanella oneidensis* MR-1 to boost electricity generation in MFC^11^; however, cisplatin can also act as an electron shuttle that depicted in cyclic voltammetry (CV) plot^13,14^. Though these physiochemical and biological approaches successfully improved electricity in bench-top MFCs, however, these are uncertain for application in full-scale MFCs, either due to high cost and not being as an eco-friendly^6^. Thus, a low-cost alternative, which could be applied to enhance EET among syntrophic partners with well-understood mechanisms is warranted.

Plant secondary metabolites (PSMs) are low-cost and can be easily extracted from various parts of plant such as leaf, seed and bark, etc. These PSMs are widely classified as tannins, saponins and essential oils, which are polyphenolic compounds having diverse molecular structures^15,16^. A few PSMs, from *Camellia sinensis (L.) Kuntze*, e.g., epigallocatechin-3-gallate (EGCG), gallocatechin, gallic acid as well as anthocyanin, have shown electron shuttling properties, which were recently shown to enhance the power density in MFCs after acclimatization for a period of 2.5 years following community enrichment^8,17^. It was ascertained that, the functional groups present in the molecules of PSM [e.g., dihydroxyl (− OH) substituents], if present in the ortho or para position of benzene ring, could significantly exhibit stable reversible electron-shuttling characteristics^8^. However, the effect of PSM on indigenous EET properties of EAMs remained unclear as there was no evidence to show the effect of PSM-based acclimation on microbial population dynamics and their EET behavior. For the first time in this research it is demonstrated that microbial cell stress associated with exposure to PSM causes alteration in cell morphology, form tannin-protein complex, and also enhances EET. This PSM-based strategy to enhance electricity production in small laboratory scale-MFC was further validated in pilot-scale MFC treating septage.

## Results

### Preliminary investigation on plant secondary metabolites

**Figure 1:**
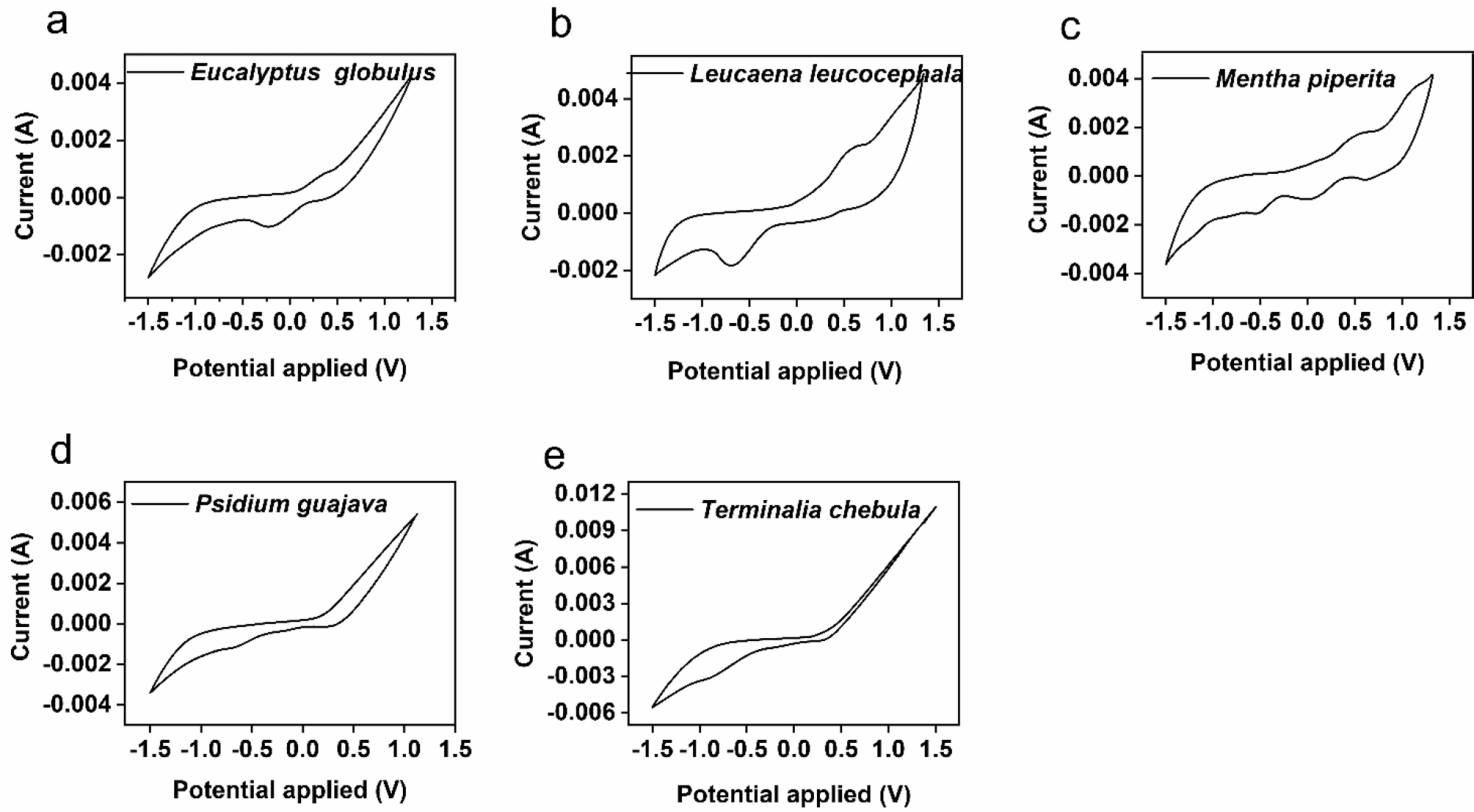
Determination of electron shuttling properties of PSM. (**a**–**e**) The cyclic voltammograms (CV) of different PWE was performed without addition of microbes, which illustrated both oxidation and reduction peaks. The closed-loop area was estimated from the CV curves (V. mA), in the order *Mentha* (4.2) > *Leucaena* (3.9) > *Terminalia* (3.7) > *Eucalyptus* (2.2) > *Psidium* (2.1). Graphs are plotted by using NOVA 1.11 software (https://metrohm-autolab.com/Products/Echem/Software/Nova.html) and Origin 9.0 software (https://www.originlab.com/).

**Figure 2:**
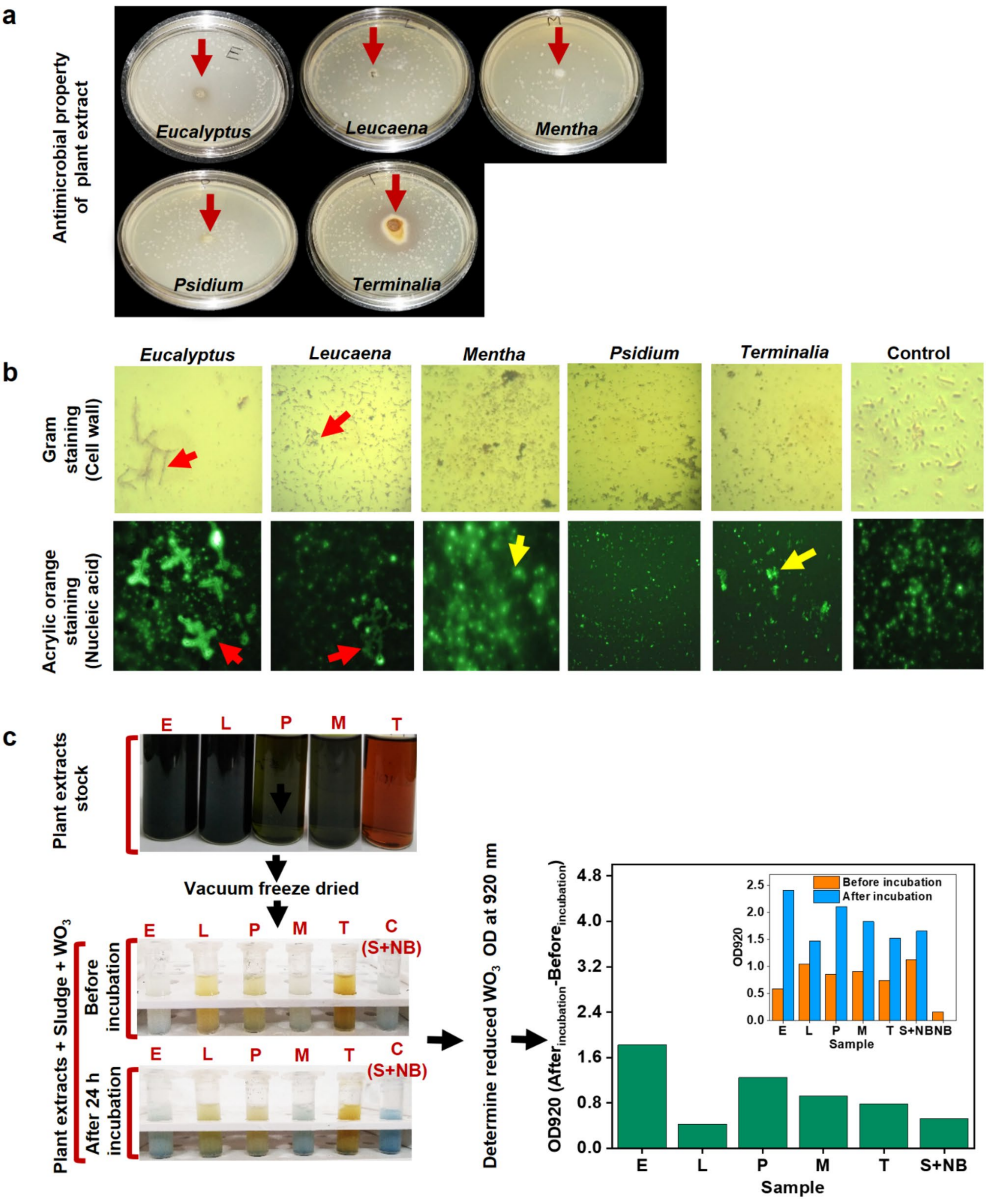
Determination of electrochromic activity and antimicrobial properties. (**a**) Antimicrobial test with all PWE-PSM-dose (1 mg of PWE per 1 ml of mixed anaerobic sludge having VSS of 25 g.l^-1^ which is ~ 10^6^ CFU per ml), the red arrows represent the location of wells on which the PWE-PSM-dose was loaded. (**b**) 1. Gram staining for bacterial cell wall, and 2. Acrylic orange staining for bacterial nucleic acid (after 24 h of incubation with various PWE-PSMs in anaerobic condition) i.e., *Eucalyptus globulus* (E), *Leucaena leucocephala* (L),* Psidium guajava* (P), *Mentha piperita* (M), *Terminalia chebula* (T) and without PSM treatment as control (C). In the case of E-dose and L*-*dose, chain formation, filamentous growth (red arrow) and spheroplast formation (yellow arrow) were visualized. In case of other PWE-PSM-dose, reduction in cell size and cluster formation was observed. (**c**) The WO_3_ based electrochromic activity test was carried out $\left[ {WO_{3} oxidized \left( {white} \right) + e^{ - } \to WO_{3} reduced \left( {blue color} \right)} \right]$ at 920 nm (OD_920_)^22^, with all PWE-PSM-dose treated mixed anaerobic sludge and detonated with respective plant species names, sludge (S) + nutrient broth (NB). Graphs are plotted by using Origin 9.0 software (https://www.originlab.com/).

Plant water extracts (PWE) of *Eucalyptus globulus* (E), *Leucaena leucocephala* (L),* Psidium guajava* (P), *Mentha piperita* (M), and *Terminalia chebula* (T) were prepared by following the ultrasonic assisted water extraction method^18^, and the presence of different phytochemicals such as tannins, saponins, and total phenolic compounds were quantified as described in Methods^19^ (Supplementary Table S1 and Table S2). The PWE-PSMs from different plant species were analysed for their electron shuttling properties, antimicrobial activity, effect on bacterial morphology and WO_3_-based electrochromic activity as per described protocols^17,20–22^ (See details in Supplementary Note S1). Results from these preliminary investigation confirmed that PWE-PSMs can act as an electron shuttle, form tannin-protein complex with bacterial cell, cause alteration in bacterial cell morphology such as cluster formation, chain and spheroplast (cell wall free cells) development depending on the source of plant species and these properties all together played key role for facilitating EET (Figs. 1a–e, 2a–c).

### Performance of MFCs inoculated with various PWE-PSM treated consortia

The preliminary results presented above thus demonstrate that PWE-PSM have inherent redox activity and can enhance EET of microorganisms present in the mixed anaerobic sludge. Later, the effect of PSM-PWE on EET in MFCs was investigated. Same mixed anaerobic microbes were exposed to all PWE-PSM (1 mg of PWE-PSM per ml of mixed anaerobic sludge consortia) and incubated for 24 h before inoculating into the anodic chamber of a MFC. The laboratory scale MFCs were operated in batch mode with acetate based synthetic wastewater as feed having chemical oxygen demand (COD) of 3 g.l^-1^ under closed circuit mode with external resistance of 100 Ω (Supplementary Fig. S1). Batch cycle of operation with 3 days feed frequency was adopted and PWE was not added later throughout the experiment^17^.

There are several investigations where it was clearly evidenced that altering the external resistance in MFC resulted in shifting of the microbial population dynamics on the anode surface^6,7^. Besides, the external resistance also played a key role in shifting between the electrogenesis and methanogenesis in mixed microbes enriched on the anode surface that led to influence the overall electricity production in MFC^7^. As in the present investigation, the main focus was to observe the effect of PSM on microbes developed on anode and their influence on electricity production in MFC, the external resistance was kept constant throughout the operation of all lab-scale MFCs and it was not altered to optimize the power production of individual MFC.

**Figure 3:**
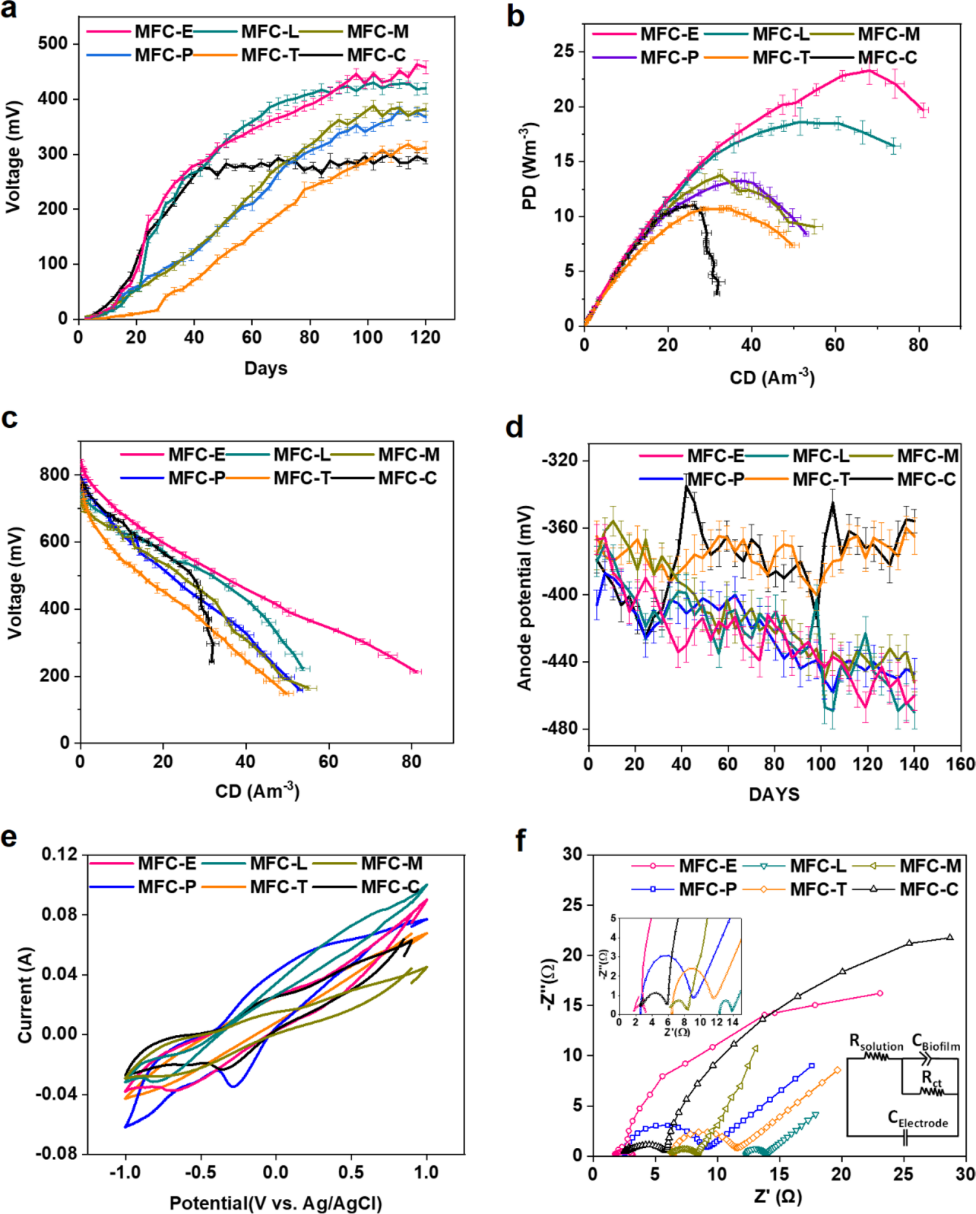
Electrical performance of all MFC-PWE_microbes_. (**a**) Operating voltage variation in MFCs with respect to 100 Ω external resistance; (**b**) power density curve (40,000–50 Ω); (**c**) polarization curve; (**d**) anode potential variation; (**e**) cyclic voltammetry of the anodic biofilm developed in MFC; (**f**) EIS spectra of MFCs. Graphs are plotted by using NOVA 1.11 software (https://metrohm-autolab.com/Products/Echem/Software/Nova.html) and Origin 9.0 software (https://www.originlab.com/).

During the first 20 days of batch operation, lower operating voltage (OV) values across 100 Ω of external resistance were observed for all five MFCs inoculated by using mixed anaerobic sludge with PWE-PSM treatment (MFC-PSM_microbes_) than the MFC-C, inoculated with mixed anaerobic consortia without any treatment (Fig. 3a). This supports the observation that PWE-PSMs have some inhibitory effect on sludge bacteria due to the presence of PSM (Fig. 2a), e.g. *Terminalia* extract contains gallic acid, chebulic acid, chebulanin, terchebulin, etc.^23–25^ and *Eucalyptus* extract has terpenes, acylphloroglucinols, euglobals, etc.^26^ and mimosine presence in case of *Leucaena*^27^. The control MFC-C achieved a steady-state after 40 days of operation, depicting a stable OV value of 292 ± 5 mV. In contrast, the OV values continued to increase steeply in case of MFC-PSM_microbes_, except the MFC-*Terminalia* (Fig. 3a).

After 80 days of operation, the OV values were still increasing for MFC-E, MFC-L, MFC-M, and MFC-P as compared to MFC-C and MFC-T (Fig. 3a) and finally the steady-state was reached in all MFC-PSM_microbes_ after 90–120 days of operation. The MFC-E produced the highest OV of 443 ± 12 mV followed by 421 ± 6 mV, 360 ± 14 mV, 372 ± 10 mV and 304 ± 11 mV in case of MFC-L, MFC-P, MFC-M and MFC-T, respectively (Table 1a). Unexpectedly, MFC-T could not attain desirable increment in OV as compared to MFC-C (292 mV ± 8) indicating this PSM is not suitable for pre-treatment of mixed anaerobic sludge to enrich EAB on anode.

**Table 1:** Electrochemical performance of MFC-PSM_microbes_ during steady state.

MFC	Max. power density during polarization		Max. current density during polarization		Internal resistance	OV _40,000 Ω_	OV _100 Ω_
	W m^-3^	mW m^-2^	A m^-3^	mA m^-2^	Ω	mV	mV
(a). Mixed anaerobic consortia as inoculum							
E	23.3	291.6	68.2	855.2	62	781	441
L	18.6	233.4	51.6	646.7	69	745	420
M	13.8	172.6	32.5	408.0	86	715	358
P	13.2	177.1	36.4	486.7	113	780	364
T	10.7	134.1	32.7	410.0	101	781	327
C	11.0	138.2	26.2	329.2	117	757	292
(b). *P. aeruginosa as* inoculum							
*PA	8.4	104.8	18.3	229.2	214	842	264
**PAE	31.8	399.1	67.4	845.5	52	847	538

MFCs were inoculated with different PWE-PSM-treated mixed anaerobic microbes (PWE such as E: *Eucalyptus globulus*, L: *Leucaena Leucocephala*, M: *Mentha piperita*, T: *Terminalia chebula*, P: *Psidium guajava)* and MFC-C: control using mixed anaerobic microbes without any PWE pre-treatment.

*MFC inoculated with *P. aeruginosa* without any PWE treatment (PA).

**MFC inoculated with *Eucalyptus globulus* PWE*-*treated *P. aeruginosa* (PAE).

An incremental pattern was obtained in OV values in MFC-PSM_microbes_ even after four months of operation, especially, in MFC-E and MFC-L (Fig. 3a). This is corroborated due to the fact that, PSM can form tannin-protein complex^28^ with bacterial proteins, which was observed as settleable fraction during WO_3_-test of different PWE-PSMs on microbes (Fig. 2c). Though it was assumed that PSMs could have undergone microbial degradation^29^ for this prolong operation; however deposition of tannin protein complex at bottom of the anodic chamber cannot be ruled out, thus ensuring effect for prolong duration of operation. The duration required to achieve stable OV in this case was still lower than the previous findings where gallotannic acid-toxicity on anaerobic microbes was persistent over 2 months (with neutral pH condition)^30^ and a period of 2.5 years acclimation of mixed microbe against tea metabolites was required to sustain the use of tea metabolites as electron shuttle for microbes and to reach stable OV^8,17^.

In tannin-protein complexes, the hydrogen bonds are formed between the tannin's phenol group and the carbonyl group of the protein's peptide^28^. Again, this is also true that, tannin-protein complex formation was irreversible at neutral pH, and can be reversible under high acidic or high alkaline pH^30^. Though it also depends upon the molecular size of tannin; e.g., the anaerobic degradation of phenolic compounds by granular sludge can take different time periods, such as for pyrogallol, gallic acid and gallotannic acid it took 2—5 days, for phenol 31 days were required and for catechol (l,2-dihydroxybenzene) it took 56 days under the same experimental conditions^30^. This is due to the fact that, the oligomeric tannins can effectively inhibit and/or induce microorganisms by binding with surface target sites; whereas, low molecular weight tannins can be penetrated inside cell wall^28^. Thus, it can be concluded that the tannin can stay for prolong period and prompt in EET associated with bacterial cells enriched in anodic biofilm of MFC, since the swarming properties of bacteria can be only affected in the presence of selective PSMs with higher concentration of dose^31^.

The maximum PD obtained during polarization of MFC-E was 23.2 W m^-3^, which was followed by MFC-L (18.6 W m^-3^) and it was 110% and 69% higher than the PD achieved by control MFC-C (11 W m^-3^), respectively (Fig. 3b,c and Table 1a). Furthermore, comparable PDs of 13.2 W m^-3^ and 13.7 W m^-3^ were observed for MFC-P and MFC-M, respectively. The lowest PD of 10.7 W m^-3^ was obtained in case of MFC-T, which was comparable to the control MFC-C (11.0 W m^-3^). The anode potential was measured to be more negative in case of MFC-E, MFC-L, MFC-M and MFC-P as compared to MFC-T and MFC-C (Fig. 3d), which could be corroborated to the high electron shuttling between EAB and anode (Fig. 3a–c); however the energy gain per electron transferred was less for production of cell biomass in case of the former MFCs^32^.

The internal resistance of MFCs was estimated from slope of the cell voltage-current curve generated during polarization and it was found that MFC-E had least internal resistance as compared to other MFCs (Table 1a). It should be noted that the preliminary exposure of microbes to *Eucalyptus*-extract exhibited clusters formation, chain formation and elongated cell structure (Fig. 2b), which was corroborated to the previous findings, where cell elongation and filamentous growth in *E. coli.* and *S. oneidensis MR-1* was observed when exposed to cisplatin (anti-cancer drug)^11^ and resulted in a five-fold increment in current density of MFC^11^.

To further investigate the anodic oxidation reactions and the charge transfer resistance (*R*_*ct*_) in the matured anodic biofilms in MFCs, the CV and electrochemical impedance spectroscopy (EIS) analyses were performed. Two oxidation peaks were detected for all the MFCs indicating EET between the microbes and the anode. The first peak was detected around − 0.8 V versus Ag/AgCl in all MFCs, except for MFC-T, where the peak was detected at—0.68 V versus Ag/AgCl (Fig. 3e). The second peak was detected around—0.08 V versus Ag/AgCl for all MFCs including MFC-C. However, in case of MFC-P and MFC-L, a shift in the positive direction was noticed, and the peaks were detected at + 0.20 V versus Ag/AgCl and + 0.34 V versus Ag/AgCl, respectively. Likewise, the charge transfer resistance for MFC-E, MFC-L, MFC-M, MFC-P, MFC-T and MFC-C was estimated to be 1.4 Ω, 1.5 Ω, 2.1 Ω, 6.1 Ω, 5.1 Ω, 3.35 Ω, respectively (Fig. 3f). Hence, it was confirmed that the exposure of PWE to the EAB and their subsequent inoculation in MFC undergo a significant alteration in their rate of EET mechanism and conductivity of anodic biofilm.

In addition, the effect of PWE-PSMs on EET components of anodic biofilm, such as cytochrome c and Type IV pili, formate dehydrogenase (*fdh*) activity, (Supplementary Fig. S2, Fig. S3 and Note S2), was evaluated. The cell polarizability and respective electrochemical activity also depends upon different cell morphology, e.g. sphere shape has the lowest polarizability than the elongated cell shapes, such as cell extensions or pseudopodia^33^. This is due to the fact that, in case of elongated cells, the cell internals including electron transport chains, membrane gradients, cellular ionic electrolytes are in a very close vicinity with final electron accepters (anode in case of MFC) and gets better opportunity for exchange of electrons as compared to the spherical cells^33^. A high *fdh* activity was observed in case of *Eucalyptus*-dose treated microbes (Supplementary Fig. S2 and Note S2) that indicated higher recycling ratio of intracellular oxidation and reduction of nicotinamide adenine dinucleotide (NAD^+^/NADH)^60^. The higher NADH concentration means higher recycling of NAD^+^ that assists the release of more number of electrons from bacterial intracellular space to extracellular components^60^. Interestingly, the *Eucalyptus*-dose caused high *fdh* activity in bacteria and also it has demonstrated highest effect on cell cluster and chain formation in microbes (Fig. 2b). This argument is the proper explanation of why the filamentous growth and long chain formation in bacterial cells have better efficiency in EET and enhanced PD in MFC.

### PCR-DGGE analysis for anodic microbiota developed in different MFCs

**Figure 4:**
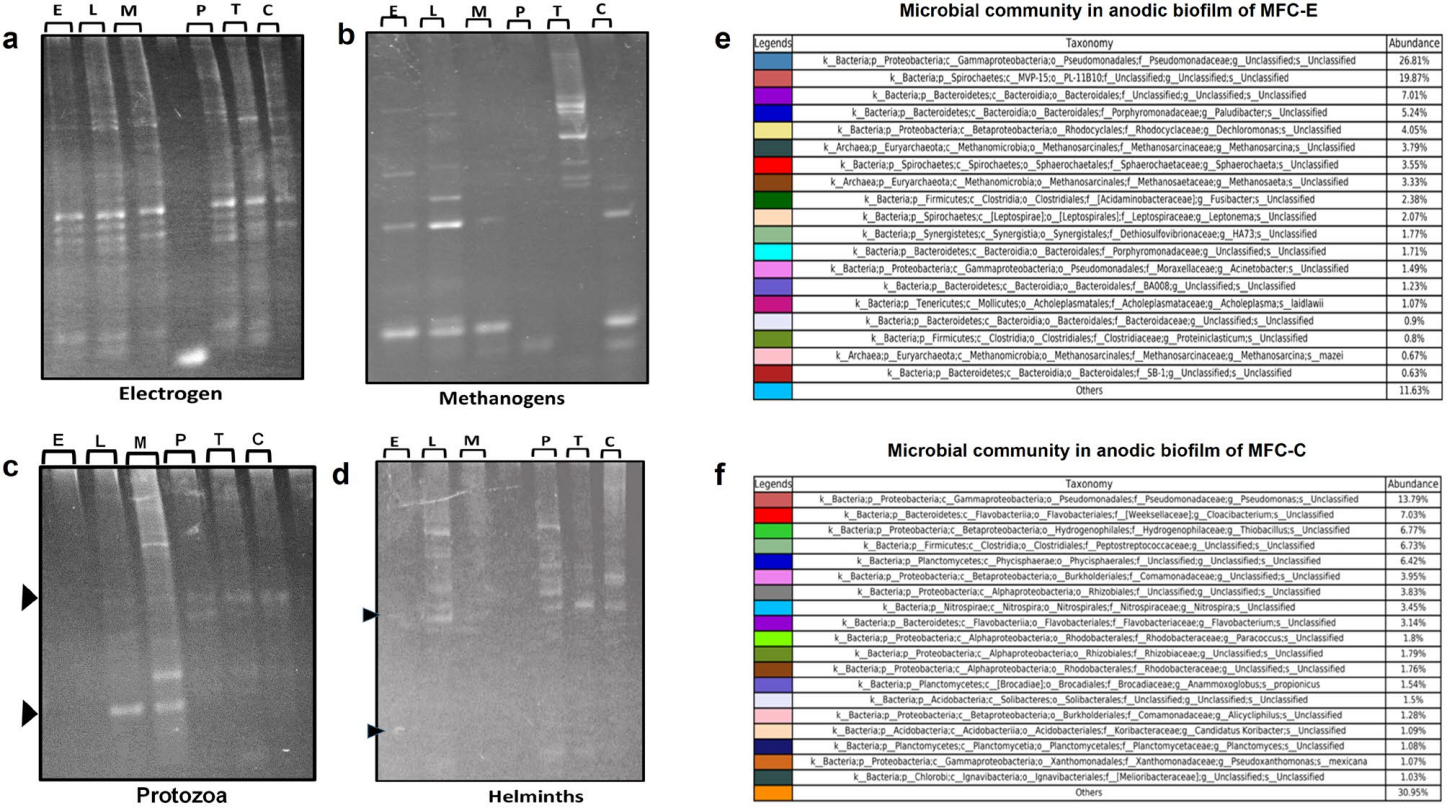
PCR-DGGE profile and NGS analysis of anodic biofilm. PCR-DGGE profile of anodic biofilm, after the end of 140 days of operation of MFCs. The respective anodic biofilms were scrapped and all the microbiota shifting was analysed through amplifying the microbes-specific primers in PCR followed by DGGE, which includes (**a**) Exoelectrogens; (**b**) Methanogens; (**c**) Protozoa; and (**d**) Helminth. The letter notations above were based on the individual PWE pre-treated mixed anaerobic sludge used as inoculum for formation of the biofilm as follows: *Eucalyptus globulus* (E)*, Leucaena leucocephala* (L)*, Mentha piperita* (M)*, Psidium guajava* (P)*, Terminalia chebula* (T) and (C) as control. The black arrows show the faint bands. Bacterial-archaea diversity in anodic mixed consortia developed in (**e**) MFC-E and (**f**) MFC-C. Gels are visualized by using Quantity One Software (https://www.bio-rad.com/en-id/product/quantity-one-1-d-analysis-software?ID=1de9eb3a-1eb5-4edb-82d2-68b91bf360fb).

The effect of PWE-PSM on microbial population shifting in anodic biofilm was analysed by polymerase chain reaction-denaturing gradient gel electrophoresis (PCR-DGGE) method using different microbes-specific primers (Supplementary Table S3). After four months of operation of MFC, shift in the exoelectrogenic group of bacterial population in anodic chamber of MFCs-PSM_microbes_ was observed (increased in band numbers, which were amplified with exoelectrogenic bacterial-primers), except in MFC-T and MFC-C where no such bands appeared (Fig. 4a). In case of methanogenic population, a few new bands appeared in case of both anodic biofilms from MFC-E and MFC-L, respectively, as compared to the control MFC-C. Whereas, few bands disappeared in case of MFC-M and MFC-P (Fig. 4b). This is a clear sign among methanogens for shifting of metabolic pathways from methanogenesis towards exoelectrogenesis (mcrA gene was down-regulated in MFC with close circuit operation)^34^, which was again agreed with the recent investigation that methanogens such as *Methanosarcina acetivorans* performed EET with Fe(III) in the presence of 2-bromoethanesulfonate (inhibitor of methanogenesis)^35^.

The band patterns for protozoal and helminth populations illustrated a significant alteration in their population dynamics in case of all PWE-treated anodic microbes (Fig. 4c,d) due to the effect of PSMs as reported earlier^36^. This shift in microbial diversity in anodic biofilm was affecting the efficiency of COD removal and coulombic efficiency obtained in MFC-PWE_microbes_ as compared to MFC-C (Supplementary Fig. S4 and Note S3).

### Next generation sequencing for MFC-E and MFC-C anodic biofilm

High-throughput sequenced 16S rRNA library was prepared for investigating anode microbial community profiling in MFC-E (MFC with highest PD), with accession number SRR12666014 (National Centre for Biotechnology Information, NCBI, USA) and compared with microbes developed in anode of MFC-C with accession number SRR12095174 (where mixed microbes from same sources without any pre-treatment and similar MFC set up was used). It was observed that, though in both cases Pseudomonas Class was dominated; however, the abundance of this class was more in case of MFC-E (26.81%) as compared to MFC-C (13.79%) (Fig. 4e,f). This could be the fact that, *Pseudomonas aeruginosa* sp. is one of the most resistance sp. against a wide range of toxic substances including antibiotics and bioactive compounds owing to its highly negatively charged phospholipid bilayer stacking of outer membrane^37^. Furthermore, the Spirochaetes Class was also present in case of MFC-E (19.87%), whereas it was absent in MFC-C. Among the Bacteroidetes class, in MFC-C, a new *Cloacibacterium* genus appeared with abundance of 7.03%; whereas, *Paludibacter* genus appeared in case of MFC-E (12.25%). In addition, considering above 2% abundance, in MFC-E, *Dechloromonas* genus was 4.05%, which belongs to Order Betaproteobacter; this was followed by genus *Methanosarcina* (3.79%), *Fusibacter* (2.38%) and *Leptonema* (2.07%).

A completely different microbial diversity profile was however identified in case of MFC-C; a new genus *Thiobacillus* appeared with abundance of 6.77%, which belongs to Order Betaproteobacter, Class Firmicutes (6.77%), Plantomycetes (6.42%), the Betaproteobacteria with Family Comamonadaceae (3.95%), Rhizobiales (3.83%) belonging to Alphaproteobacteia Class, *Nitrosprira* (3.45%), and *Flavobacterium* (3.14%). All these microorganisms are EABs and can produce electricity in MFC^1,4^. Though a complete diversity of bacterial population was identified in between MFC-E and MFC-C; however, their respective power output was way different and could not be directly correlated. Therefore, the effect of *Eucalyptus*-extract dose on pure culture of *P. aeruginosa* was further investigated in MFC.

### Optimization of Eucalyptus-dose and evaluating effect on pure culture of P. aeruginosa and electrochemical performance in MFC

#### Optimization of dose of *Eucalyptus* extract for mixed anaerobic microbes

In the aforementioned investigations, it was confirmed that the *Eucalyptus*-dose (1 ml of *Eucalyptus* extract per ml of anaerobic sludge having VSS of 25 g.l^-1^, equivalent to 10^6^ CFU.ml^-1^) was effective on mixed anaerobic microbes for resulting the highest PD in MFC; however, the response of microbes depends on the optimum dose. In an earlier investigation, it was reported that, tannin-dose from *Carob pod* could be able to initiate filamentous growth in *Pseudomonas fluorescens,* nonetheless not in case of *Escherichia coli*^38^. Hence, mixed anaerobic consortia was preferred for the determination of optimum dose of the *Eucalyptus*-extract. The *Eucalyptus*-extract dose was selected in an increasing order as 0.5 mg, 1.0 mg, 1.5 mg, 2.0 mg and 5.0 mg per 1 ml of anaerobic sludge having VSS concentration of 25 g.l^-1^ and named as E_0.5_, E_1.0_, E_1.5_, E_2.0_, and E_5.0_, respectively. The sucrose medium with 20% sucrose and 0.15% MgSO_4_.7H_2_O in glucose-basal medium^38^ was used for inoculating the mixed bacteria by keeping the incubation temperature at 30 ˚C.

**Figure 5:**
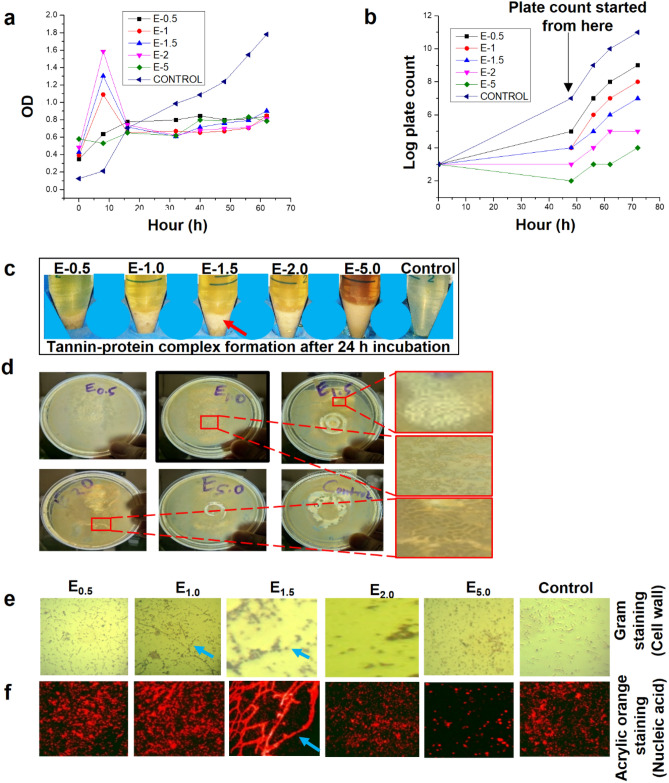
Response of mixed microbes treated with *Eucalyptus* extract –PSM and optimization of E-dose (**a**) Tannin-protein complex formation after 24 h with increasing order of *Eucalyptus*-dose (indicated by red arrow). (**b**) Effect of increasing order of *Eucalyptus* extract-dose on the viability of mixed microbiota represented by plate count (CFC per ml) after 24 h of incubation at 30 ˚C. (**c**) Represent different morphological growth of microbes, wrinkle and ring structures on petri-plates with increasing order of *Eucalyptus*-dose. (**d**) The pale yellow colour pictures are Gram stained microbes and the red colour pictures are nucleic acid stained by acryl orange. The change in cell morphology, filamentous growth and spheroplast formation are displayed with respect to different concentrations of *Eucalyptus*-doses (0.5 mg, 1.0 mg, 1.5 mg, 2.0 mg and 5.0 mg) per 1 ml of sludge with VSS of 25 g.l^-1^. (**e**) Gram staining of mixed microbes (for cell wall). (**f**) Acrylic orange staining for nucleic acid. Graphs are plotted by using Origin 9.0 software (https://www.originlab.com/).

The mixed anaerobic culture was incubated with different *Eucalyptus*-dosage. Cell growth and cell counts were performed through OD_600_ and plate count methods as previously described^38^ keeping a time interval of 8 h. It was observed that there was a sudden rise in the OD_600_ values in culture medium with E_1.0_, E_1.5_, and E_2.0_ as compared to the untreated microbes and other E-dosages (Fig. 5a). This sharp increment in OD_600_ values with respect to the increasing concentrations of *Eucalyptus*-dose is due to the rapid lysis of some of the bacterial cells and release of cytoplasmic fractions into the medium. Unexpectedly, in case of E_5.0_–dose, bacterial growth was slower (Fig. 5b). There was no evidence of such sharp peaks in OD_600_ values, probably due to the high concentration of E_5.0_–dose, rapid lysis of bacterial cells might have occurred and subsequently settled down along with tannin-protein complex at the bottom (Fig. 5c, presented with red arrow). This tannin-protein complex formation is a rapid phenomenon and hence might have occurred within the time period of 8 h (as 8 h interval was used for inspecting the samples). However, after 10—20 h the OD_600_ values reduced significantly owing to the formation of tannin-protein complex^39^ (Fig. 5c, cream yellow fraction at the bottom) and subsequently settled down at the bottom of culture tubes.

The colony forming unit (CFU) count were also performed for per ml of microbes incubated with different *Eucalyptus*-dosage (Fig. 5b). The CFU.ml^-1^ count depicted that, the *Eucalyptus*-dose has inhibition effect on the microbial growth with increasing order of concentrations of dose as compared to the untreated microbes. Similarly, some unusual microbial growth on petri-plate was observed with increase in *Eucalyptus*-dose (Fig. 5d). Remarkably, the microscopic observation confirmed a unique growth pattern, especially the spheroplast and filamentous and chain formation in case of E_0.5_, E_1.0_ and E_1.5_ (Fig. 5e,f). Higher *Eucalyptus*-dose (i.e. E_2.0_ and E_5.0_) significantly inhibited the CFU counts and the persisted cells turned into small sizes or unusual shapes. Pore-like lesions and ring structure were observed around the cells, indicating the leakage of cytosolic fluids during drying process caused by extreme damage of bacterial cell^40^. This response of mixed microbes is also in agreement with the previous investigation, where tannin exposure led to morphological changes, irregular rod formation with wrinkled surfaces and altered the total cellular fatty acids, remodel FapC fibrils and turned into amorphous aggregates in *E. coli*^41,42^ and caused grooves in *P. aeruginosa*^40^ or propagate as L-forms (cell with without cell wall)^43^ against cisplatin, ciproflixcin and prolong starvation^11,44^. Hence, E_1.5_-dose (1.5 mg of *Eucalyptus*-dose per ml sludge) is considered as the optimum for creating external stimuli on mixed consortia for higher EET.

### Effect of *Eucalyptus*-dose on *P. aeruginosa* and evaluating electrochemical performance in MFC

**Figure 6:**
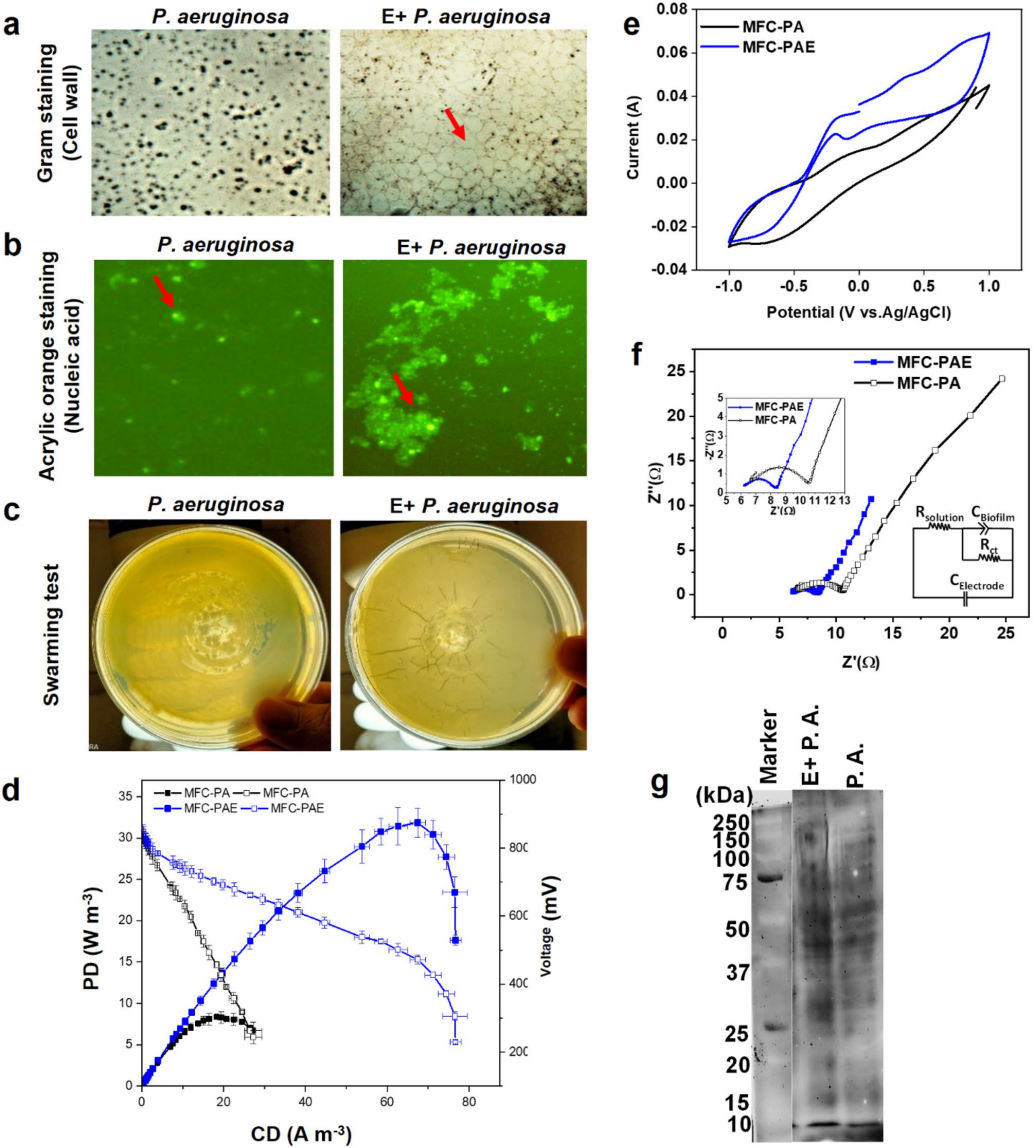
Effect of E_1.5_-dose on *P. aeruginosa* and performance in MFC. (**a**) Petri plate growth of *P. aeruginosa* with and without E-treatment; (**b**) Polarization and power density curves of MFC, without E-treated *P. aeruginosa* (PA) and with E-treated *P. aeruginosa* (PAE) as inoculum; (**c**) CV profile; (**d**) EIS spectra; and (**e**) Heme staining of anodic biofilm developed by E-treated PA and PA. Graphs are plotted by using NOVA 1.11 software (https://metrohm-autolab.com/Products/Echem/Software/Nova.html) Origin 9.0 software (https://www.originlab.com/) and Quantity One Software (https://www.bio-rad.com/en-id/product/quantity-one-1-d-analysis-software?ID=1de9eb3a-1eb5-4edb-82d2-68b91bf360fb).

As *Pseudomonadaceae* was observed to be the most dominating genus in the anodic biofilm of MFC-E, hence, *P. aeruginosa* was selected as reference EAB for investigating the effect of *Eucalyptus* dose (E_1.5_) on EET. Pure culture of *P. aeruginosa* was incubated with E_1.5_-dose for 24 h followed by inoculation in anodic chamber of MFC. Alteration in the cell morphology and formation of ring-like structure in the *P. aeruginosa* culture treated with E_1.5_-dose (E + *P. aeruginosa*) was observed; whereas, there was no such change in the untreated *P. aeruginosa* (Fig. 6a, Gram-staining). This result was validated through acrylic orange staining, where close aggregates or cluster formation was visualized with clear evidence of spheroplast (Fig. 6b), which was very similar to the previous findings that tannic acid caused cluster formation and filamentous structure in *Pseudomonas protegens* pf-5^45^.

However, the effect of E-tannins and polyphenols on the *P. aeruginosa* was mild as compared to mixed culture (Fig. 2a), which depicted the strong tannin-resistance properties of *P. aeruginosa* as previously described^31^. The swarming motility and the growth pattern of *P. aeruginosa* on the petri plate was verified, which depicted that the E_1.5_-dose has no such inhibition on swarming zone in *P. aeruginosa* as compared to the control, i.e. untreated *P. aeruginosa* (Fig. 6c). This result also confirmed that the optimum E_1.5_-dose has no adverse effect on the quorum sensing mechanism of *P. aeruginosa* for biofilm formation^31^.

Furthermore, E_1.5_-treated *P. aeruginosa* was inoculated in anodic chamber of MFC (MFC-PAE) and untreated *P. aeruginosa* inoculated in MFC-PA was considered as control. The maximum PD of 31.0 W m^-3^ was achieved in MFC-PAE, which was 3.8-times higher than PD of 8.0 W m^-3^ achieved by MFC-PA (Fig. 6d; Table 1b). The measured OCV values of 860 mV and 852 mV were observed in MFC-PAE and MFC-PA, respectively (Supplementary Table S4). The higher OCV obtained in case of MFC-PA is due to the fact that *P. aeruginosa* can accumulate more charge as compared to the mixed culture and other EABs^46^. The measured anode potentials were -302 mV and -299 mV and cathode potentials were 558 mV and 553 mV in MFC-PAE and MFC-PA, respectively (Supplementary Table S4).

The CV analysis depicted two oxidation peaks at -0.72 V and -0.79 V vs Ag/AgCl in case of MFC-PA and MFC-PAE, respectively (Fig. 6e). However, the second peak region was shifted towards the negative side to -0.09 V for MFC-PAE as compared to potential of + 0.34 V for MFC-PA. These results clearly depicted that, the EET was significantly carried out through endogenous mediators such as pyocyanin secreted from *P. aeruginosa*^47^. The anodic current and PD were significantly higher for MFC-PAE as compared to MFC-PA. Likewise, the charge transfer resistance for MFC-PAE was found to be 2.1 Ω, which was lower than that for MFC-PA (4.4 Ω) (Fig. 6f). This is attributed to the effect of E_1.5_-dose. The Heme-staining of whole cell lysate experiment also supported that the heme proteins (including c-type cytochromes) in *P. aeruginosa* were not affected with E_1.5_-dose (Fig. 6g), and hence E_1.5_-dose is suitable for inducing EET in both pure stain as well as mixed consortia.

### Application of E_1.5_-dose in pilot-scale MFC

**Figure 7:**
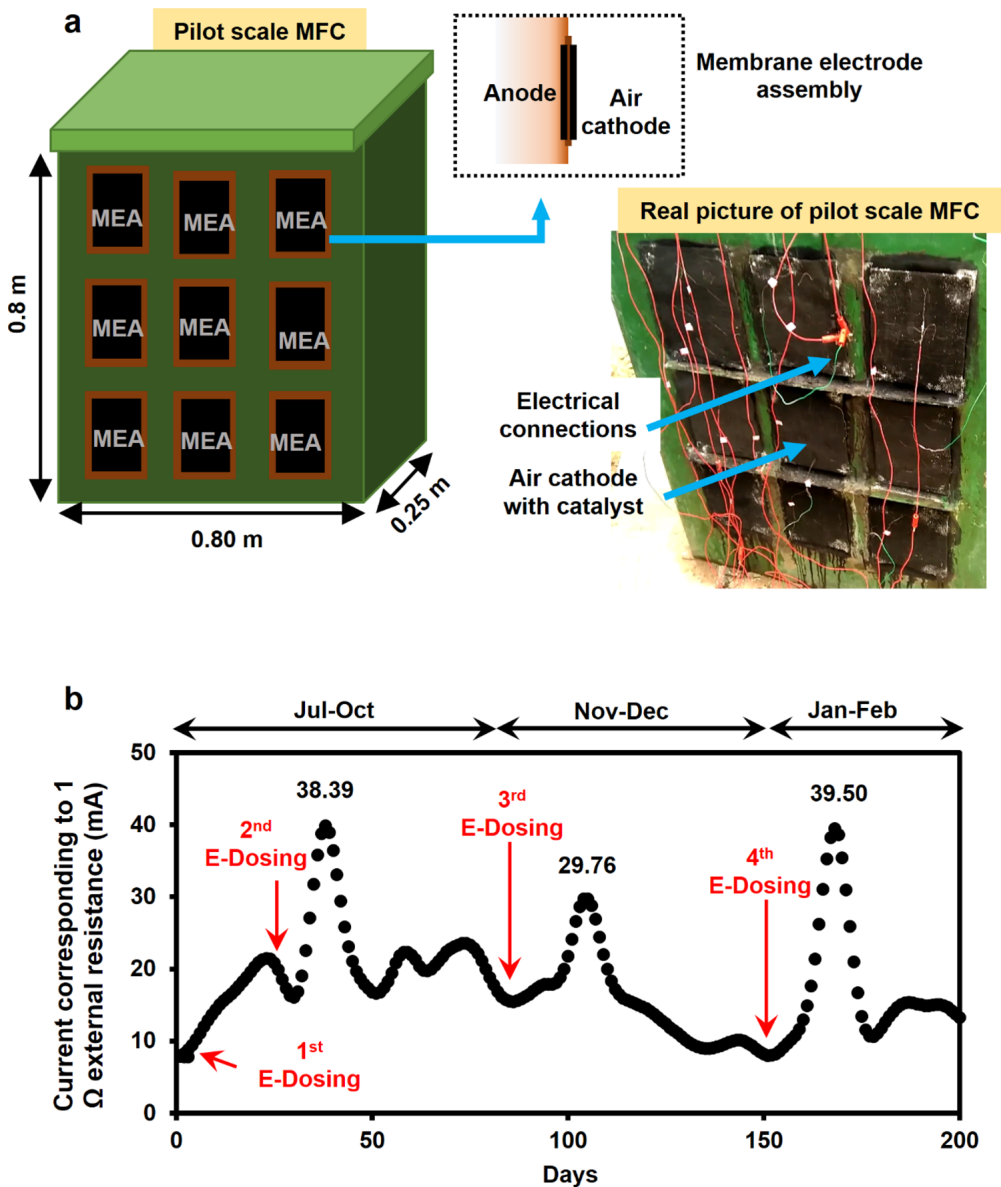
Demonstration of pilot-MFC with *Eucalyptus*-dosing. (**a**) Schematics of pilot-scale air cathode MFC having 125 l of anodic chamber volume. (**b**) Current production profile of pilot scale MFC. The operating current (OC) value was recorded with respect to 1 Ω. Graphs are plotted by using Origin 9.0 software (https://www.originlab.com/) and Microsoft PowerPoint (https://www.microsoft.com/en-in/microsoft-365/microsoft-office?rtc=1).

A field-scale air cathode MFC having anodic chamber volume of 125 l and consisting of 9 pairs of anode-membrane-cathode assembly was installed and operated with septic tank slurry (COD = 2685 ± 960 mg.l^-1^) collected from septic tanks of IIT Kharagpur campus (Fig. 7a). This pilot-scale MFC (pilot-MFC), was inoculated with approximately 40 l of sludge having VSS of 25 g.l^-1^. The PWE of *Euccalyptus* with a dose of 6.0 l was applied to 40 l of anaerobic sludge in the anodic chamber of pilot-MFC. The 2^nd^, 3^rd^ and 4^th^ E_1.5_-dosing cycles were performed on 30^th^, 90^th^ and 150^th^ days of operating period of pilot-MFC, respectively. In each E_1.5_-dosing cycles, approximately 18.75 l of *Eucalyptus* leaves-extract was added to the anolyte volume of 125 l of pilot-MFC.

During initial days of start-up period of pilot-MFC after giving first E_1.5_-dose, a gradual increment pattern in operating current (OC) value was observed with respect to external resistance of 1 Ω (Fig. 7b), which indicated the acclimation period of electroactive bacteria on anode. After applying the 2^nd^ E_1.5_-dose*,* sudden drop in OC value was recorded for one day and then it raised up to 38.39 mA during subsequent days. The first OC-peak was lasted over a period of 15–20 days. We waited for further enhancement in OC values without addition of E_1.5_-dose, however no further significant increment in current was observed up to 90 days, then the 3^rd^ and 4^th^ E_1.5_-dosage were applied to anolyte of pilot-MFC on 90th and 150th days of operation (Fig. 7b). Here, again an increase in the OC was noticed. This results depicted that, E_1.5_-dose can easily boost the electricity production of pilot-MFC, which corroborated as the electron shuttling properties, tannin-protein complex formation and alteration in cell morphology (Figs. 2c, 5c–f) are the major cause for higher EET mechanism in sludge microbes. However, a decrease in the OC value was observed between 110 and 150 days of operation, which could be due to the seasonal variation in temperature during months of November and December, where the ambient temperature reached to around 10–12 ˚C under Indian condition.

In the 125 l pilot-scale MFC, after the initial start-up time the PD of 4.0 mW m^-2^ was obtained for 160 days of stable phase of operation. A maximum PD of 83 mW m^-2^ was reported earlier in MFC with working volume of 85 l; however after one month of operation the performance was reduced^48^. Though the PD was higher in case of 85 l MFC as compared to our case, however in another investigation, air–cathode MFC with a volume of 255 l of anodic chamber, it was noticed that PD was 43.2 mW m^-2^ during initial 14–35 days of operation that gradually decreased to 8.5 mW m^-2^ on 56–77 days of operation^49^.

Similarly, the present investigation pertaining to the operation of 125 l pilot-scale air cathode MFC, the CE of 2.2% was obtained before addition of PWE to the anodic inoculum; however, after addition of E_1.5_–dose to anodic inoculum, CE value was increased to a maximum of ~ 5% (with respect to 1 Ω external resistance). However, in the past investigation on MFCs with anodic chamber volume of 85 l (assembled with 22 anodes) and 255 l prototypes, CE values of 22% and 29.5% were observed, respectively. However after a close investigation, it was observed that in case of MFC with volume of 255 l, the CE value which was around 29.5% (14–35 days), got decreased to 4.5% (36–56 days), 2.4% (56–77 days), and to 3.8% on 77 to 98 days of operation of pilot-scale MFC (with respect to 2.5 Ω external resistance), which is comparable to CE obtained in present investigation with 125 l MFC, where CE of around 5% was observed. This difference in the CE values among the three different sizes of MFCs was due to the use of application of different types of electrode materials, ORR catalysts, proton exchange membrane, and different configuration and volume of the anodic chamber used.

## Discussion

We investigated preliminary experiments to analyze the characteristics of PSMs and their effects on microbes. It was revealed that PSMs with different molecular structures are substituted with diverse functional groups that signify with unique electrochemical properties. Also, in case of leaves, the photosynthesis process is carried out in a series of electrochemical reactions, where the electrons flow assisted via several electron carriers such as plastoquinone, plastocyanin ferredoxin, etc. Hence, it might be possible that PSM from leaves could have additional electron shuttles for assisting EET in bacteria.

The operating voltage of MFC-PWE_microbes_ illustrated that, PSMs have a long term effect on EABs with a single spiking and short pre-incubation period of 24 h. This is the first investigation to demonstrate regarding this long term effect of PSMs in MFC. This long term electrochemical properties of PSMs in MFC is due to the strong tannin-protein complexes formation^50,51^ with bacterial cells present in the inoculum. These tannin-protein complexes can be formed by three types of bonds such as (1) hydrogen bonds (e.g., between the carbonyl oxygen or the amido nitrogen of the peptide bond, the phenolic hydroxyl of the tannin and free amino groups in protein, or the hydroxyl and carboxyl groups of other polymers), (2) ionic bonds or salt linkages, and (3) covalent bonds (due to the conversion of phenols to quinones or semi-quinones, which can bind a large groups of protein^50^.

This is also true that, tannins can be degraded by anaerobic microbes^38^, however tannin-toxicity can sustain up to several days to several months^30^. In the previous investigation, it was illustrated that, condense tannins (e.g., epigallocatechin-3-gallate (EGCG), gallocatechin, gallic acid) facilitate electron shuttling and enhanced PD in MFC^52,53^. Nevertheless, both hydrolyzable tannins and condensed tannin form tannins-protein complex depending upon pH conditions. The hydrolyzable tannins bind through hydrogen bonds via un-ionized carboxyl or carbonyl groups, whereas the condensed tannin bind through the phenolic hydroxyls^50,51^. Hence, in this investigation it is proposed that both hydrolyzable and condensed tannin present in the plant extracts have key role to play in the long term enhanced performance of MFC.

In our investigation, though PWE-PSMs from different plant species contained tannins; however, their molecular structures and size determines the binding efficiency on the target sites of bacterial cell. Smaller size PSM can penetrate in the bacterial cell and larger sizes bind with the cell membrane structure^28^. Hence, depending on the binding of PSMs at different target sites of bacterial cells that result in activating and/ or deactivating certain bio-molecular pathways^15,16^, which includes inhibition of cell division and form elongated cells^38,45^. These cell elongation and highest cluster formation in microbes was demonstrated when exposure to *Eucalyptus*-dose and these results are correlated with the enhancement in activity during WO_3_-electrochromic activity test (Fig. 2b,c) and the electrochemical activity in MFCs (Fig. 3a–d). This is also corroborated with the previous finding that, cisplatin, carob pod and other chemicals such as antibiotics were able to inhibit cell division that resulted in induced elongation and filamentous growth in bacteria^11,38,40–42,44^ and the addition of cisplatin to *shewanella oneidensis* MR-1 led to increment in PD in MFC^11^.

These cisplatin-induced elongated cells demonstrate a unique growth pattern; spiral and twisted arrangement of cells on anode surface as compared to normal shaped cells and this distinct adaptation behaviour of the elongated cells resulted in covering maximum anode surface and higher EET^11^. Hence, in case of *Eucalyptus* PWE treatment, long cluster formation was observed with the bacterial cells. It is speculated that one side of the elongated bacterium hooked up to the anode leaving the other end in a hanging condition, such that it could cover less area and allow more bacteria to attach on anode for maximum electricity production in MFC^11^. The elongated form of bacteria can also take the advantages of long-distance EET to the anode as cable bacterium does, which could reduce the chance of electron loss in case of mediator-based EET.

It is noteworthy to mention that, elongated cells have better electrical properties than spherical ones, which was correlated to polarizability in the bacterial cell envelop^54^. Hence, this cell polarizability concept is also correlated with our investigation regarding *Eucalyptus*-dose based cell elongation and higher electrochemical activity in bacterial cell. This higher electrochemical activity was also observed in anodic biofilm of MFC-E with higher *fdh* activity that maintains intracellular NAD + /NADH couple and induces higher electron flux, where *Eucalyptus* PWE dose treated mixed microbes were inoculated (Supplementary Fig. S2). This is corroborated with previous investigation that, cells with spheroplast condition stretches the periplasm containing the respiratory chain system (cell elongation), including c-type cytochromes, which can efficiently perform EET without any adverse effect^55,56^.

Similarly, PSMs also possess cytotoxic properties (e.g. mutagenic, antifungal, antiviral, and antibacterial)^15,16^. The PCR-DGGE and NGS investigation revealed that PWE-PSM dose caused shifting in microbial population dynamics including exoelectrogens (bacteria), methanogenic archaea, etc., in different anodic biofilms, that tempted us to rethink about the correlation between shifting of microbial dynamics and their contribution for electrochemical properties in MFCs. Though later it was confirmed that *Eucalyptus* –PWE dose (E_1.5_) can even be able to enhance the power performance of MFC inoculated with pure culture of *P. aeruginosa*. The band distribution in DGGE experiment displayed a clear shifting in protozoa and helminth composition in the anodic biofilm. Though at this time there is very few evidences to show the harmful effect of protozoa^57^ and helminth^58^ on the performance of MFC; however, this investigation creates a scope to develop a PWE-based strategy to control the population of protozoa and helminth in an eco-friendly manner.

Furthermore, E_1.5_-dose was applied in anolyte of pilot-scale MFC, and a significant enhancement of current production was noticed in different dosing cycles, which is ascertained to electron shuttling properties^8,17^, formation of tannin-protein complex^30^, effect on bacterial cell morphology^11^ and shifting of microbial population dynamics. As the *Eucalyptus*-dose is composed of the mixture of several secondary metabolites, hence the effect on microbes and the improved electrochemical properties can be considered as the synergistic action of several metabolites.

## Conclusion

A low-cost PWE-PSMs-based strategy was explored for both mixed microbes as well as pure EAB strain, which demonstrated the evidence of tannin-protein complex formation and chain formation, shifting in microbial population, electron shuttling properties, etc., that all together enhanced power density in both bench-scale and pilot-scale MFCs. The *Eucalyptus* PWE–dose to mixed anaerobic microbes and pure culture of *P. aeruginosa* enhanced PD of MFC by 2.1 times and 3.8 times than their respective control MFCs operated without PSM-dosing. We were also able to enhance operating current in a pilot-scale MFC treating septage by applying *Eucalyptus*–dose (E_1.5_) to the inoculum composed of mixed anaerobic microbes. Hence, significant enhancement in electricity generation and EET in mixed anaerobic microbiota is possible by using low-cost PWE-PSM dosing, which is eco-friendly being natural compound. Hence, these low-cost PWE-PSMs opened a new door way to enhance electricity production in MFCs to achieve higher EET in EABs that too in eco-friendly manner by using natural compounds, thus holding a great opportunity for application in other bio-electrochemical systems and electro-microbiology research.

## Materials and methods

### Preparation of plant secondary metabolites

Leaves from four different plant species, i.e. *Eucalyptus globulus*, *Leucaena leucocephala*, *Mentha piperita*, *Psidium guajava* and dry fruits from *Terminalia chebula* were collected, washed, dried at 50 ˚C in a hot air oven and grinded with a mechanical grinder. The grinded product was sieved through mesh number 60 to obtain fine powder. The PWE was prepared by ultrasound-assisted extraction (UAE) method with water as the solvent using an ultrasonic cleaner equipped with a temperature controller^18^. Finally, the extracted samples were filtered through Whatman No. 1 filter paper and freeze-dried (FD5512 Flore-model, Ilshinebiobase, South Korea) and about 0.1 g (dry weight) of solid plant extracts was obtained from 1 g of powder. For all the experiments 1.0 mg of solid PWE was administered for 1 ml of anaerobic septic tank sludge (VSS = 25 g.l^-1^ or ~ 10^6^ cells CFU counts per ml) and incubated at 30 °C for 24 h and used as an inoculum in the anodic chamber of MFC. Quantification of tannin, total phenol and saponin were carried out through the previously described procedure^19^.

### Electrochromic, antimicrobial activity and cyclic voltammetry investigation for anaerobic microbes pretreated with PWE

The antimicrobial activity of different PWE dosage was evaluated with agar well diffusion method^20^. Gram staining kit and Acridine orange stain was used to stain bacterial cell wall and nucleic acid, respectively. The microscopic pictures were captured through Motic AE31 Inverted 100X phase oil immersion objective lens Phase Contrast Fluorescence Microscope (Leica Microsystems, India). The electrochromic activity test for EAM was conducted by tungsten oxide (WO_3_) based detection method^21^. Five culture tubes filled with 0.5 ml of the PWE treated sludge from respective plant extracts and one additional tube as control was kept inside the anaerobic chamber with glove box (Bactron, Sheldon Manufacturing Inc., USA). Each culture was further dosed with 2.5 ml of sodium lactate minimal salt medium and 0.012 g of WO_3_ followed by proper mixing and incubated at 37 ˚C for 30 min. The colour developed in respective PWE treated culture-vials was observed and compared with the standards. The turbidity changes in OD_600_ of mixed microbiota was administered with the increasing order of *Eucalyptus*-dose and the effect due to the alteration in growth rate of bacterial cells was estimated by subtracting the OD_600_ value of respective *Eucalyptus*-dose without microbes from OD_600_ of mixed microbiota after inoculation.

### Fabrication of MFC

All the lab-scale MFCs were fabricated with baked clayware ceramic cylinder having working volume of 100 ml and wall thickness of ~ 5 mm, in which wall of the cylinder acted as a proton exchange membrane^59^ (Figure S1). Carbon felt, stitched with stainless steel wire to work as current collector, was used as both anode and cathode having projected surface area of 80 cm^2^ and 153 cm^2^, respectively, covering the inner and the outer surface of the cylindrical ceramic pot. Anode and cathode were connected with external resistance of 100 Ω. Tap water was used as catholyte and aerated externally (Resun Electro Magnetic Air Pump ACO-003). Five MFCs were operated with different PSM treated sludge with volume of 10 ml as inoculum; MFC-E (*Eucalyptus globulus*), MFC-L (*Leucaena Leucocephala*), MFC-M (*Mentha piperita*), MFC-P (*Psidium guajava*), MFC-T (*Terminalia chebula*) along with MFC-C as control operated without any pre-treatment of plant extracts given to the mixed anaerobic sludge used as inoculum. Synthetic acetate based wastewater having COD of 3000 mg.l^-1^ and pH of 7.2 was used as feed^60^. All the MFCs were operated under fed-batch mode keeping 3 days of fresh feed frequency and operated at 30 ˚C by controlling the temperature using water heater (RiSheng, China). For undertaking experiments on evaluation of effect of PWE dose on pure culture of *P. aeruginosa* (MTCC 12,307), it was procured from Microbial Type Culture Collection and GenBank, IMTECH, Chandigarh, India.

The pilot scale air cathode MFC with anodic chamber volume of 125 l was fabricated using fibre reinforced plastic material. One side of this MFC had nine openings, which were mounted with nine membrane-electrode assemblies (MEAs)^60^. Clayware ceramic membrane (20 cm × 23 cm) was sandwiched between a carbon felt anode (15 × 18 cm) and a carbon felt cathode (15 cm × 18 cm) coated with CuSn on air facing side as cathode catalysts (at loading rate of 2 mg.cm^-2^ of cathode surface area)^61^. The septage (35 l.day^-1^) was supplied to anodic chamber of pilot-scale MFC after screening the floating matters.

### Electrochemical analysis and data acquisition

Cyclic voltammetry analysis was carried out using an electrochemical workstation (AUTO LAB AUT58696, Metrohm, Netherlands) for studying the electron shuttling characteristics of each PWE. A three electrode system was used, where the working, counter, and reference electrodes were carbon felt electrode (1.0 cm^2^), platinum electrode, and Ag/AgCl, respectively. The scanning rate of 10 mV s^−1^ was adopted over the selected voltage range of + 1.5 to − 1.5 V vs Ag/AgCl. Area of redox potential curves under the closed CV loop was determined via MATLAB 8.0 as per Eq. (1).

1 $${\text{Area}} = \mathop \smallint \limits_{{V_{L} }}^{{V_{H} }} \left( {I_{H} - I_{L} } \right) dV$$

The voltage (*V*) and current (*I*) of all the MFCs were regularly monitored using a digital multimeter having data acquisition unit (Agilent Technologies, Malaysia). Where, *V*_*H*_ and *V*_*L*_ denote the CV scanning voltage of + 1.5 V and − 1.5 V and *I*_*H*_ and *I*_*J*_ represent the oxidation current and reduction current at corresponding scan voltage, respectively. The Coulombic efficiency (CE) was estimated as per Eq. (2).

2 $${\text{CE}} = \frac{{M\mathop \smallint \nolimits_{0}^{t} Idt}}{{{\text{Fb}}v\Delta COD}}$$

where *M* denotes the molecular weight of oxygen, *I* is the current, *ΔCOD* is the substrate consumed over a batch cycle, F is Faraday's constant, *b* is the electrons generated during each mole of substrate oxidation (4 mol e^-^ per mole COD) and *v* is the anodic chamber volume.

Polarization was carried out by gradually decreasing resistance from 40,000 to 50 Ω in steps. Anodic half-cell potential was measured with respect to Ag/AgCl reference electrode (CH Instruments, Inc., RE-5B; + 0.197 V vs standard hydrogen electrode, SHE, USA) placed inside the anodic chamber. Power (*P*) was expressed as *P* = *I* * *V*. Power density was normalized to the surface area of anode or net liquid volume of anodic chamber. Anodic half-cell potential was monitored using a potentiostat (AUTO LAB AUT58696, Metrohm, Netherlands). The EIS was performed by applying frequencies ranging from 100 kHz to 100 MHz, and the electrical equivalent circuit^62^ was designed for best fitting with the observed EIS data using NOVA 1.11 software.

### PCR-DGGE fingerprinting and community diversity analysis

The anodic biofilm from each of the respective MFC was scratched separately and washed with nano-pure water. The whole genomic DNA was extracted with Power Soil DNA Isolation Kit (MoBio Laboratories Inc., Carlsbad, CA, USA) and quantified through Nano Drop spectrophotometer (Thermo Scientific, USA) following the manufacturer's instructions. PCR amplifications of the highly variable V1–V3 regions of the prokaryotic (methanogens^63^ and electrogens^64^) and the V4 region of the eukaryotic (protozoa^57^ and helminth^58^) 16S rRNA genes was conducted by PCR (Biorad T100-Thermal cycler) by maintaining specific reaction conditions (Supplementary Table S3). The DNA purity of individual microbes was verified and visualized on a 1.0% agarose gel before the DGGE assessment. The denaturing gradient gel electrophoresis was performed in DGGE-4001 system (CBS Scientific Company Inc., Del Mar, CA, USA) at 60 ˚C and filled with TAE (1X) buffer solution (40 mM Tris, 20 mM sodium acetate, 1 mM EDTA, pH 7.4). The PCR amplicons (30 μl) from each sample was loaded on polyacrylamide gel 8% (w/v) (1.0 mm thick) containing formamide (40% (w/v) and urea (7 M) denaturant gradient of 30% – 70%. Initially, a potential of 150 V was applied for 20 min and subsequently the temperature was programmed separately for individual microorganism (exoelectrogen, methanogen, protozoa and helminth). After each run, the samples were subsequently stained with ethidium bromide (0.5 µg.ml^-1^) and visualized under the Dcode Universal Mutation System (Bio-Rad) and band patterns were analysed with Quantity One Software (Bio-Rad Laboratories).

### Next-generation sequencing of anodic biofilm developed in MFC-E

The Next-generation sequencing (NGS) was carried out by preparing 16S metagenomics Sequencing Library using two PCR steps and two purification steps. Initially, 16S rRNA F (GCCTACGGGNGGCWGCAG) and 16S rRNA R (ACTACHVGGGTATCTAATCC) primer pair was used with 1^st^ amplicon to amplify the template Amplicon (size ~ 460 bp) out of a DNA sample. Synthesized primers pairs were used for specific region of interest V3-V4 with overhang adapters followed by PCR Clean Up using AMPure XP beads. The 2^nd^ PCR (Index PCR) was operated with the attachment of dual indices and Illumina sequencing adapters using the Nextera XT. The quantity and quality check of library were analysed in 4200 Tape Station system (Agilent Technologies) using D1000 Screen tape as instructed in the manual. The Sequence Read Archive was submitted to NCBI.

### SDS-PAGE and heme staining for heme-containing cytochromes

The sodium dodecyl sulfate base polyacrylamide gel electrophoresis (SDS-PAGE) and heme staining of proteins from the biofilm EPS matrix were conducted as earlier described^65^. The EPS matrix of biofilms was scraped off from the anode surface and collected in reaction buffer, followed by centrifugation at 13,000* g* for 10 min. The biofilm pellet was suspended in 1/5 volume of TNE (10 mM Tris–HCl at pH of 7.5, 100 mM NaCl, 5 mM EDTA) and further vortexed for 1 min. The SDS (0.1% weight/vol) solution was added and was mixed thoroughly at room temperature for 5 min. The sample was then passed through an 18-gauge needle followed by centrifugation at 15,500* g* for 20 min and the insoluble sheared biological fraction was collected. The pellets were collected, washed 5—7 times to remove any SDS and suspended in 10 mM Tris–HCl buffer (pH of 7.5). Then, a predetermined amount of 20 µg EPS protein (Bicinchoninic Acid protein assay) was boiled in SDS sample buffer for approximately 10 min before loading onto a 12% Mini-Protean Tris–Glycine extended gel (Bio-Rad) and a potential of 250 V was applied for 30 min. Precision Plus Protein Dual Colour Standards were used for molecular weight estimation. *N*,*N*,*N*,*N*-tetramethylbenzidine was used for staining heme-containing proteins and visualized on Gel Doc EZ Gel Documentation System**.**

### Raman spectral analysis for cytochrome detection

The biofilm coated anodes were cut and transferred into 50 ml phosphate buffered saline (PBS) solution^66^. Raman spectral analysis was carried out using micro-Raman spectrometer at a wavelength of 488 nm. Argon-Krypton mixed ion gas laser was used as a light source for excitation. The spectrometer was equipped with an optical microscope (Model BX 41, Olympus, Japan), single monochromator (Model T64000, Jobin Yvon Horiba, France), an edge filter and a Peltier-cooled CCD (1024 × 256 pixel, Model Synpse-1024X, Jobin Yvon, Horiba, France) detector. The spectral acquisition was obtained at an integration time of 0.2 s to obtain high-contrast resonance spectra for c-type cytochromes.

### Experimental studies for detecting Type IV pili by Fourier-transform infrared spectroscopy analysis

The pili expression was carried out by Fourier-transform infrared spectroscopy (FTIR) based spectral analysis as previously described^67^. Biofilm was scratched from anode of MFCs and washed gently, suspended in distilled water till the final OD (650 nm) value reached nearly 10. Then it was transferred onto the ZnSe windows, and dried to form transparent bacterial films. The FTIR spectra scans were obtained between 4000 and 650 cm^−1^ with 6 cm^−1^ spectral resolution and 64 scans using a Spectrum One FT-IR spectrometer (Thermo fisher scientific instruments**,** USA). The pili expression can be visualized by comparing the peaks especially ranging from 1600 to 1700 cm^−1^ with the literature. To investigate the changes in secondary structures, the Gaussian curve-fitting method^68^ was adopted. The PeakFit 7.2 software was used to measure the FTIR spectra of Amide I region and Origin 9.0 software was used to calculate the ratio of different types of protein secondary structure according to the integrated areas.

### Formate dehydrogenase activity (NADH/NAD^+^***)***

The metabolic activity of biofilm developed in each MFC was determined in terms of *fdh* activity catalyzed reaction^60^: +NAD^+^ → CO_2_ + NADH + Na^+^. The reaction mixture system (1 ml of each 1.67 mmol l^−1^ NAD^+^, 100 mmol l^−1^ of β-mercaptoethanol, 167 mmol l^−1^ of sodium formate and 10 mmol l^−1^ of phosphate buffer with pH of 7.5) was used for this procedure. The *fdh* activity was detected as per the described protocol^69^ using a T80 UV/VIS spectrophotometer (PG Instruments Ltd., UK) recording the change in nicotinamide adenine dinucleotide (NADH) absorption at 340 nm (εNADH = 6220 M^−1^ cm^−1^).

## Supplementary information

Supplementary Information 1.
