# Supplementary material for: Plant secondary metabolites induced electron flux in microbial fuel cell: investigation from laboratory-to-field scale
Source: Sci Rep. 2020 Oct 14;10:17185. doi: 10.1038/s41598-020-74092-y (PMC7560832; doi:10.1038/s41598-020-74092-y)
Supplement: Supplementary file 1 — Supplementary Information 1. [file 41598_2020_74092_MOESM1_ESM.docx]

**Supplementary Information**

**Plant secondary metabolites induced electron flux in microbial fuel cell: Investigation from laboratory-to-field scale**

**Dibyojyoty Nath^1^ and M. M. Ghangrekar^1,2*^**

*^1^ School of Environmental Science & Engineering, Indian Institute of Technology Kharagpur, Kharagpur-721302, India.*

*^2^ Department of Civil Engineering, Indian Institute of Technology Kharagpur, Kharagpur-721302, India.*

* Corresponding author:

Dr. M. M. Ghangrekar

Professor of Environmental Engineering

Department of Civil Engineering

Indian Institute of Technology Kharagpur

Kharagpur-721302, India

Email: [*ghangrekar@civil.iitkgp.ac.in*](mailto:ghangrekar@civil.iitkgp.ac.in)

Ph: +91-3222-283440

##
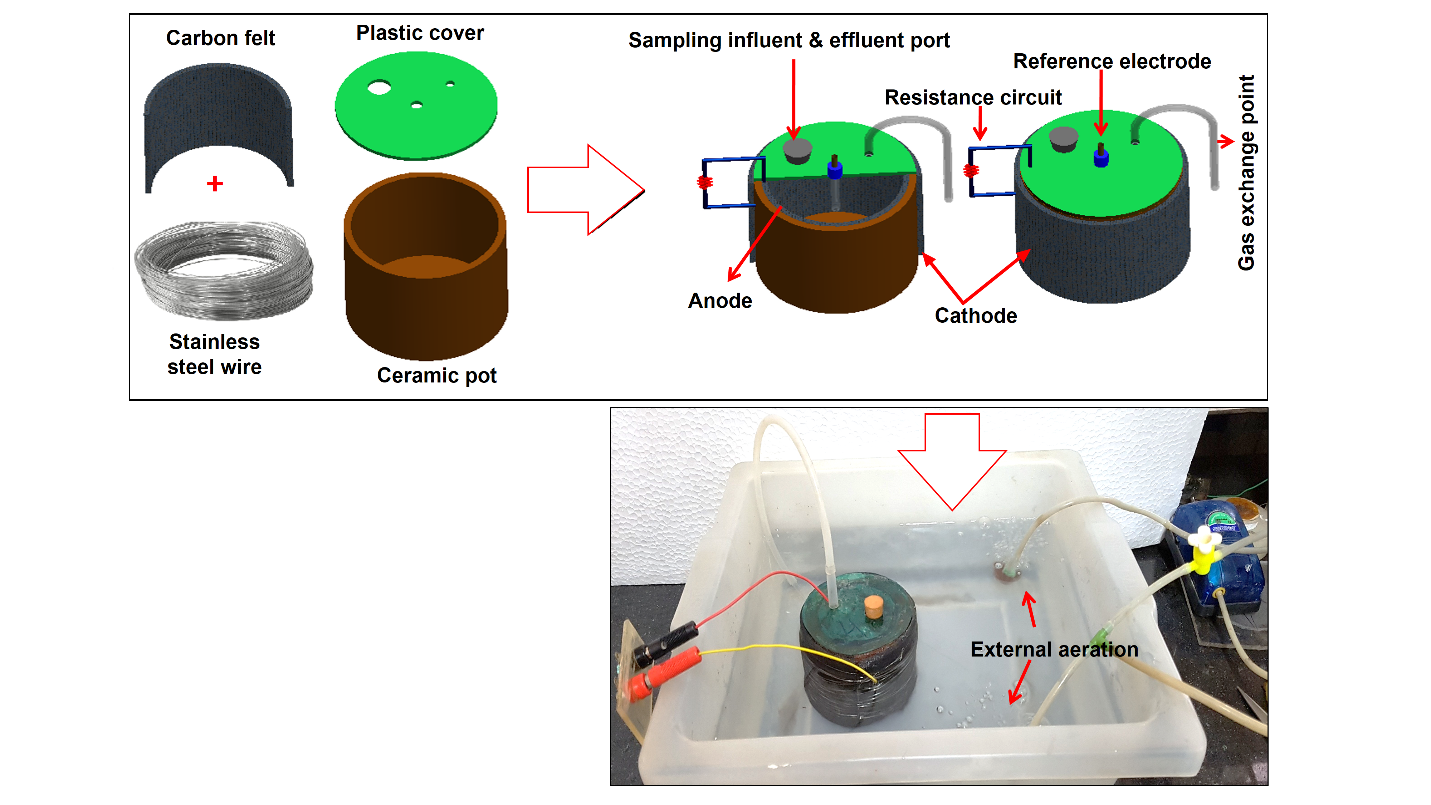


Figure S1. Detailed materials and their assembly for the microbial fuel cell. Schematics showing MFC fabricated with baked clayware ceramic cylinder having a working volume of 100 ml, in which wall of the cylinder acted as a proton exchange membrane^1^. Carbon felt with stitched stainless steel wire (as a current collector) was used as both anode and cathode. The opaque plastic cover was provided over the anodic chamber for sealing having defined ports for substrate addition or feed extraction after the end of the cycle and insertion of reference electrode. Completely fabricated MFC after assembly (right of the red arrow), anode and cathode were connected with 100 Ω resistance. The MFC operated with aerated aqueous cathode mode (bottom red arrow). Images are drawn using **Pro/Engineer software (**https://www.ptc.com/en/products/creo/pro-engineer).

##
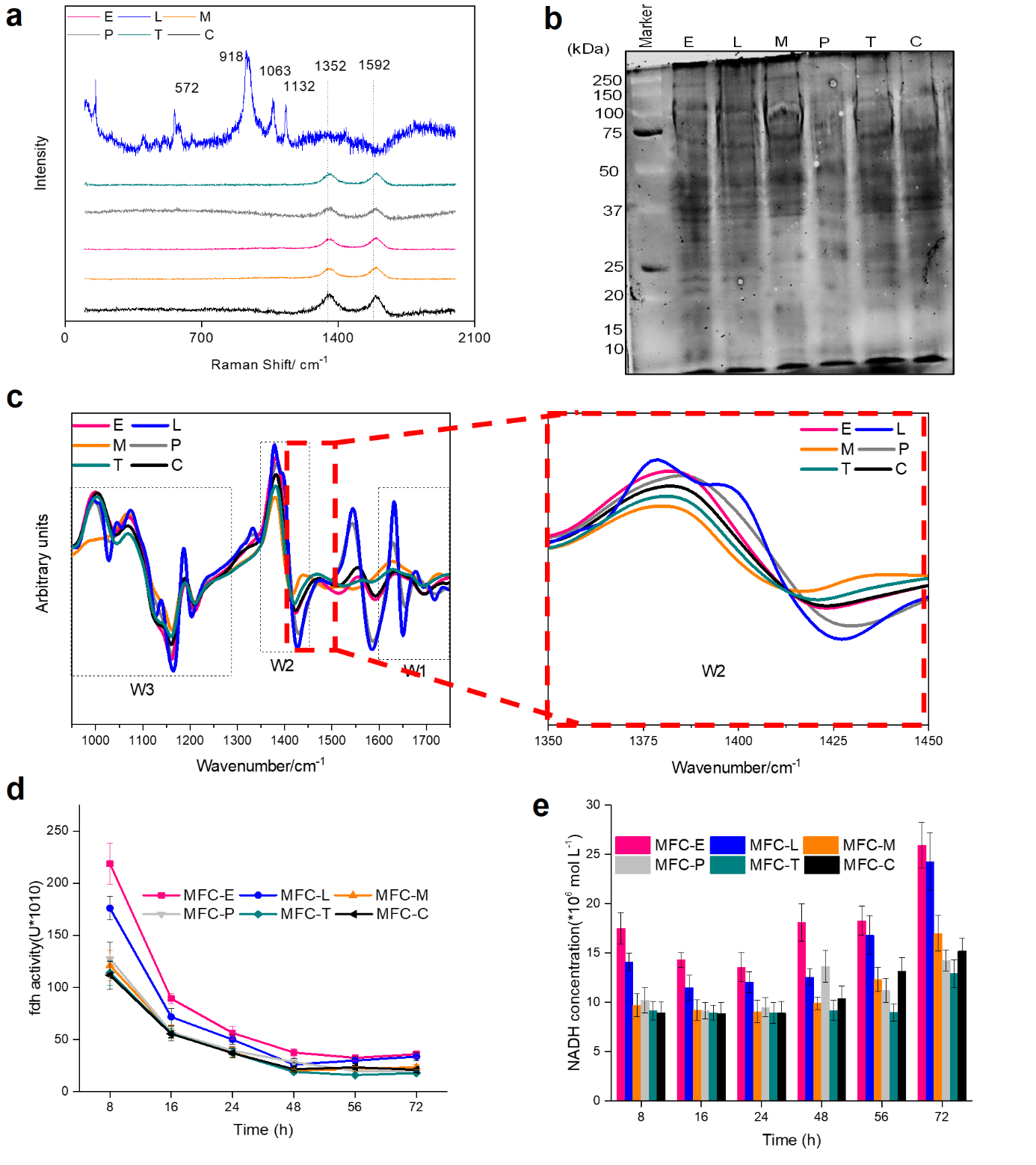
Figure S2. **Stress-induced response evaluation of EET related mechanisms in anodic biofilms.** (a) Raman spectral analysis of Cyt-c; (b) Heme staining whole cell lysate for identification of heme containing loosely bound outer membrane proteins, Cyt-c; (c) FTIR microspectroscopy absorption bands (in the range of 1800–700 cm^-1^) for Type-IV pili activity in anode biofilms; (d) NAD^+^-dependent *fdh* activity through NADH generation was represented among the anodic biofilm; (e) *fdh* activity among anodic biofilms. Graphs are plotted by using Origin 9.0 software (https://www.originlab.com/) and Heme staining gels are visualized by using Quantity One Software (https://www.bio-rad.com/en-id/product/quantity-one-1-d-analysis-software?ID=1de9eb3a-1eb5-4edb-82d2-68b91bf360fb).

##
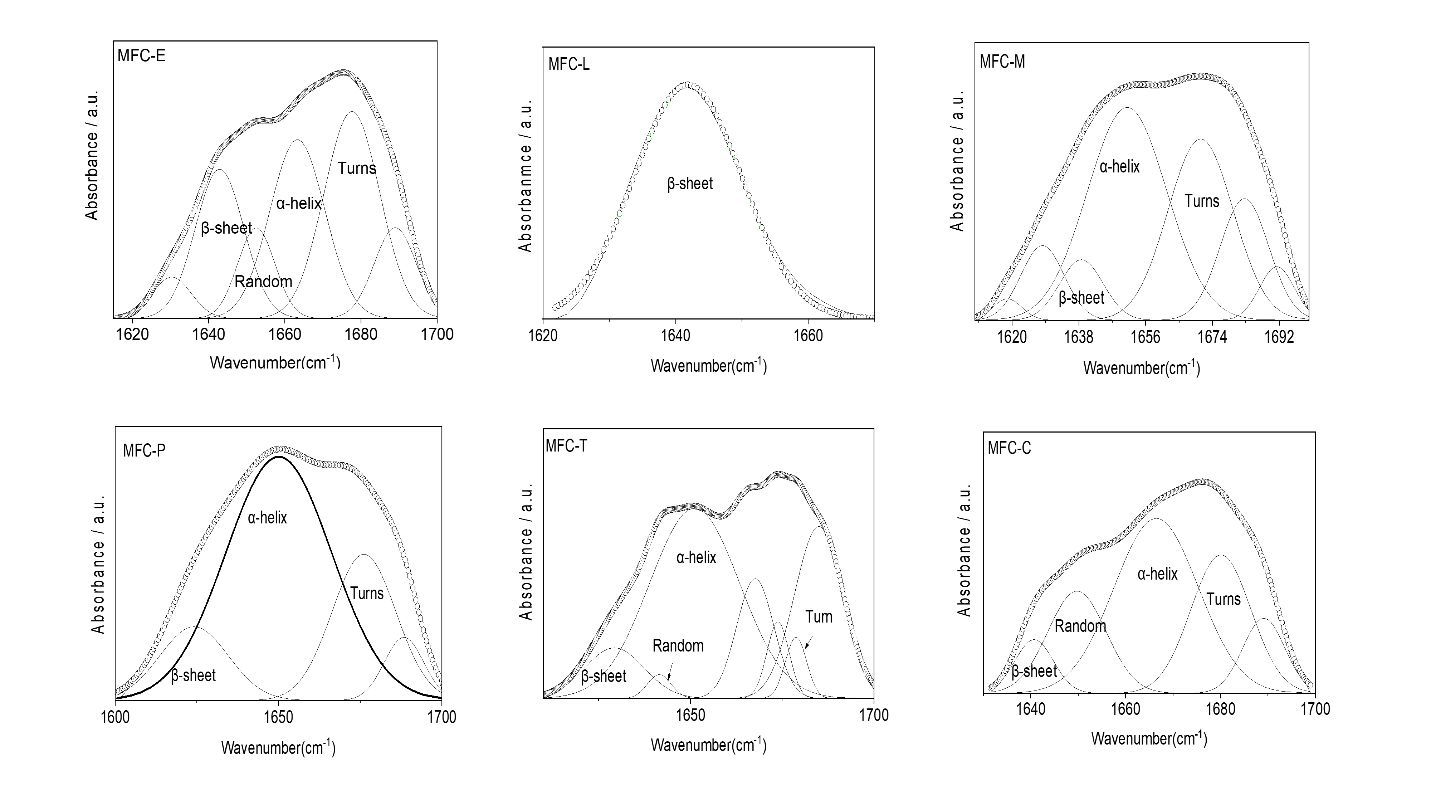


## Figure S3. Deconvolution of FTIR peak for Amide I region **in different anodic biofilms**. Deconvolution of FTIR peak for Amide I region (1600 cm^-1^ to 1700 cm^-1^) for different biofilm developed in MFCs. The area covered under the curves is presented in Table S5, which represents the corresponding protein secondary structure. Graphs are plotted by using Origin 9.0 software (https://www.originlab.com/) and PeakFit 7.2 software (https://systatsoftware.com/products/peakfit/) was used for fitting of differnet peaks.

## .

**
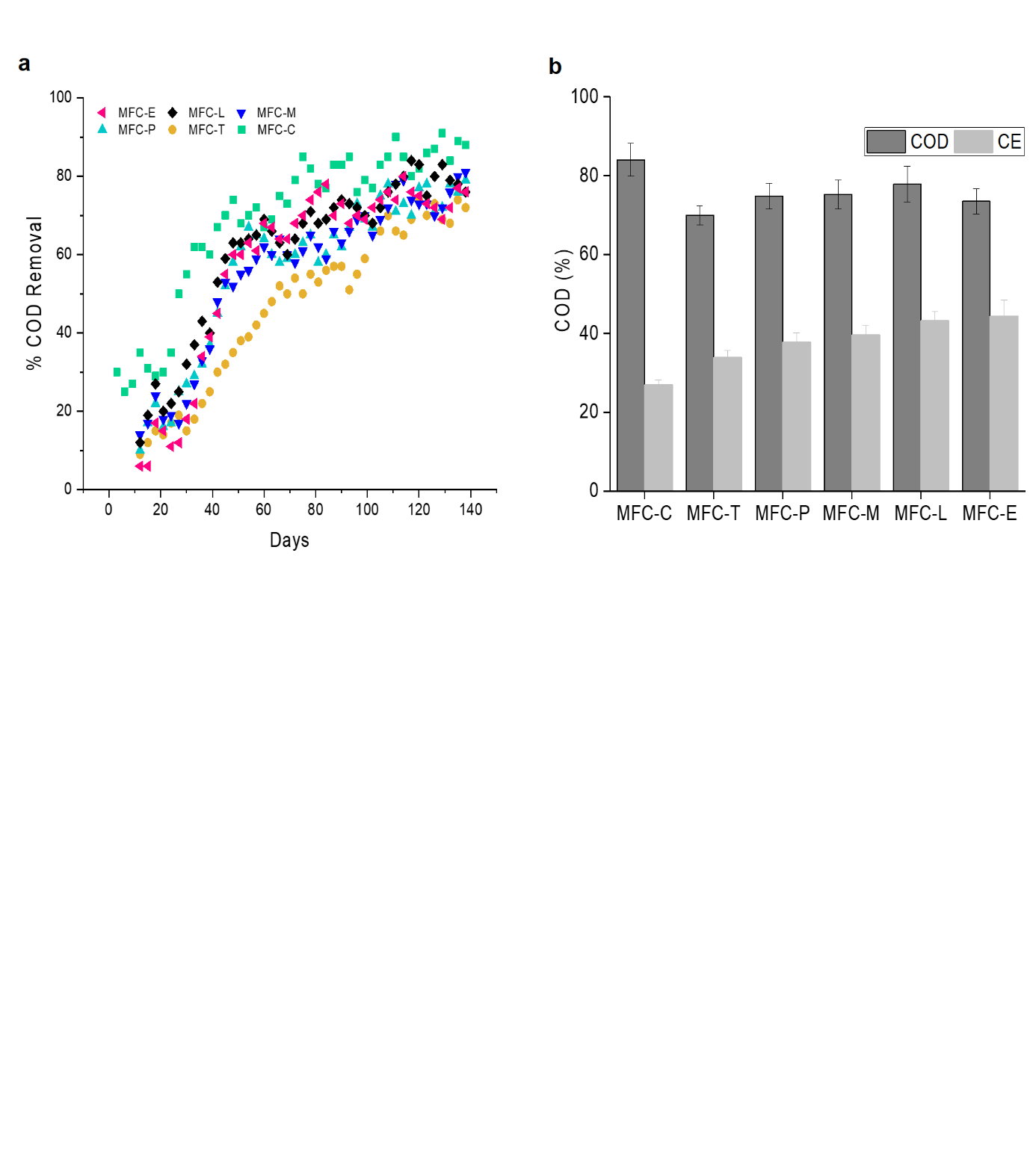
**Figure S4. COD removal and coulombic efficiency (CE) of different MFCs inoculated with PWE pretreated microbes. (a) The value inside the figure is the COD removal efficiencies of respective PWE treated MFC (influent COD = 3000 mg.l^-1^). (b) The COD removal efficiency and their respective CE values during the steady-state operation of MFC inoculated with respective PWE treated mixed microbes. The COD removal in case of the control MFC was the highest, whereas the COD removal of all the MFC-PWE_microbes_ were slightly affected, however the CEs of these MFCs were higher than control. Graphs are plotted by using Origin 9.0 software (https://www.originlab.com/).


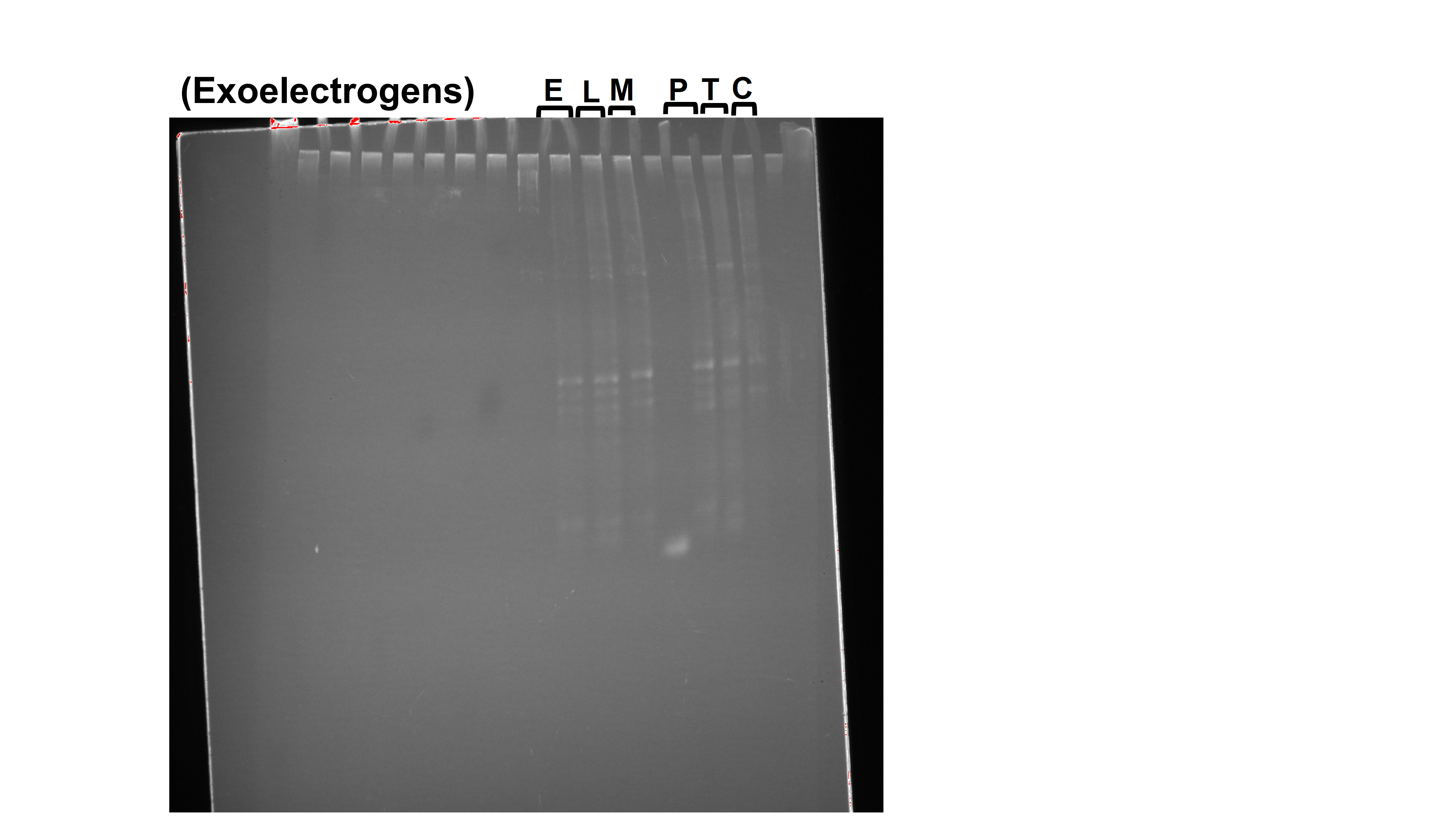


Figure S5. Original DGGE-Gel bands showing population of **exoelectrogens.** The anode biofilm developed in MFCs inoculated with different PWE-treated sludge; Eucalyptus globulus (E), Leucaena leucocephala (L), Mentha piperita (M), Psidium guajava (P), Terminalia chebula (T) and (C) as control, representing mixed anaerobic sludge without PWE treatment. Image is visualized by using Quantity One Software (https://www.bio-rad.com/en-id/product/quantity-one-1-d-analysis software?ID=1de9eb3a-1eb5-4edb-82d2-68b91bf360fb).


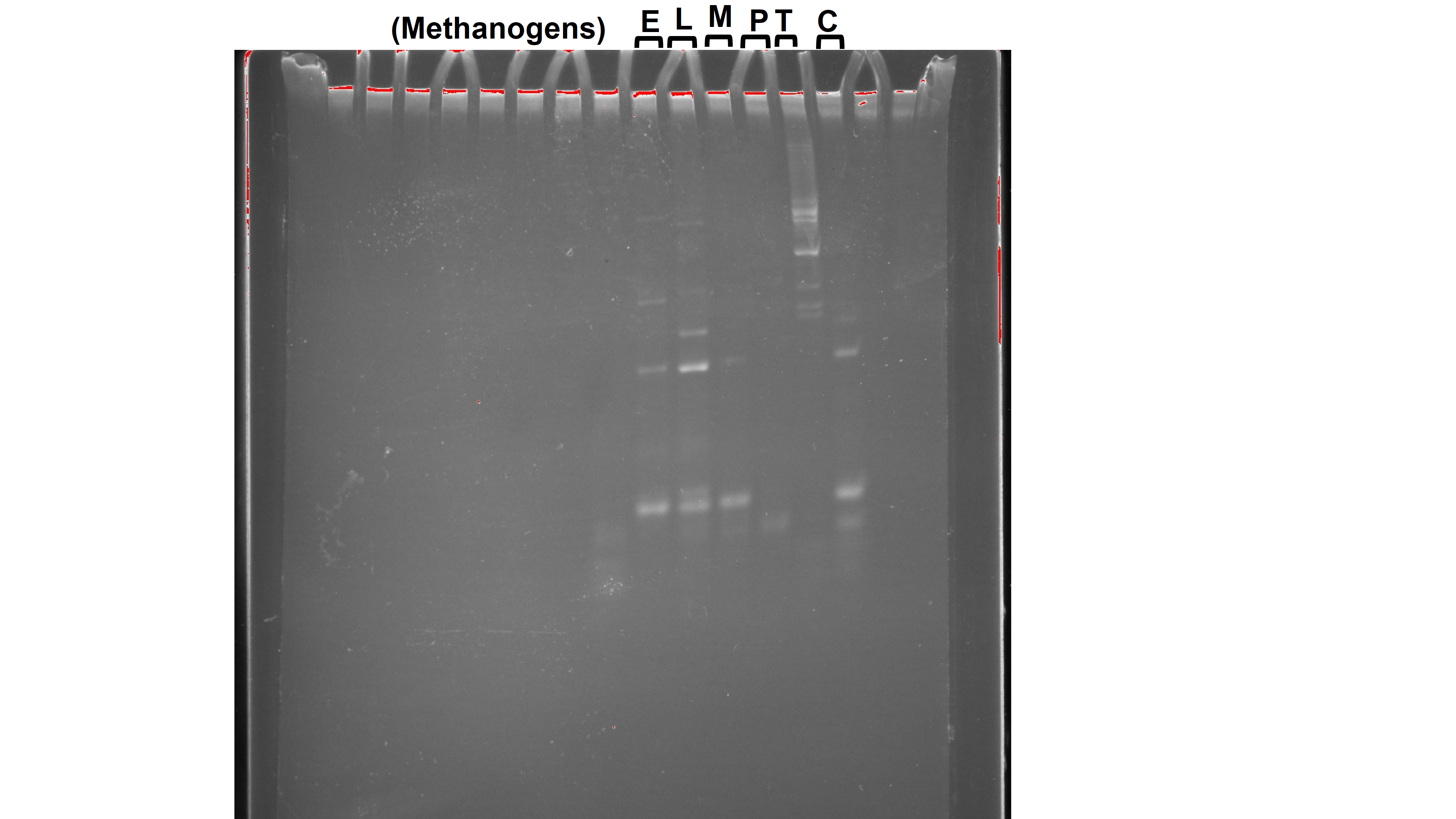


Figure S6. Original DGGE-Gel bands showing population of **methanogens.** The anode biofilm developed in MFCs inoculated with different PWE-treated sludge; Eucalyptus globulus (E), Leucaena leucocephala (L), Mentha piperita (M), Psidium guajava (P), Terminalia chebula (T) and (C) as control, representing mixed anaerobic sludge without PWE treatment. Image is visualized by using Quantity One Software (https://www.bio-rad.com/en-id/product/quantity-one-1-d-analysis software?ID=1de9eb3a-1eb5-4edb-82d2-68b91bf360fb).


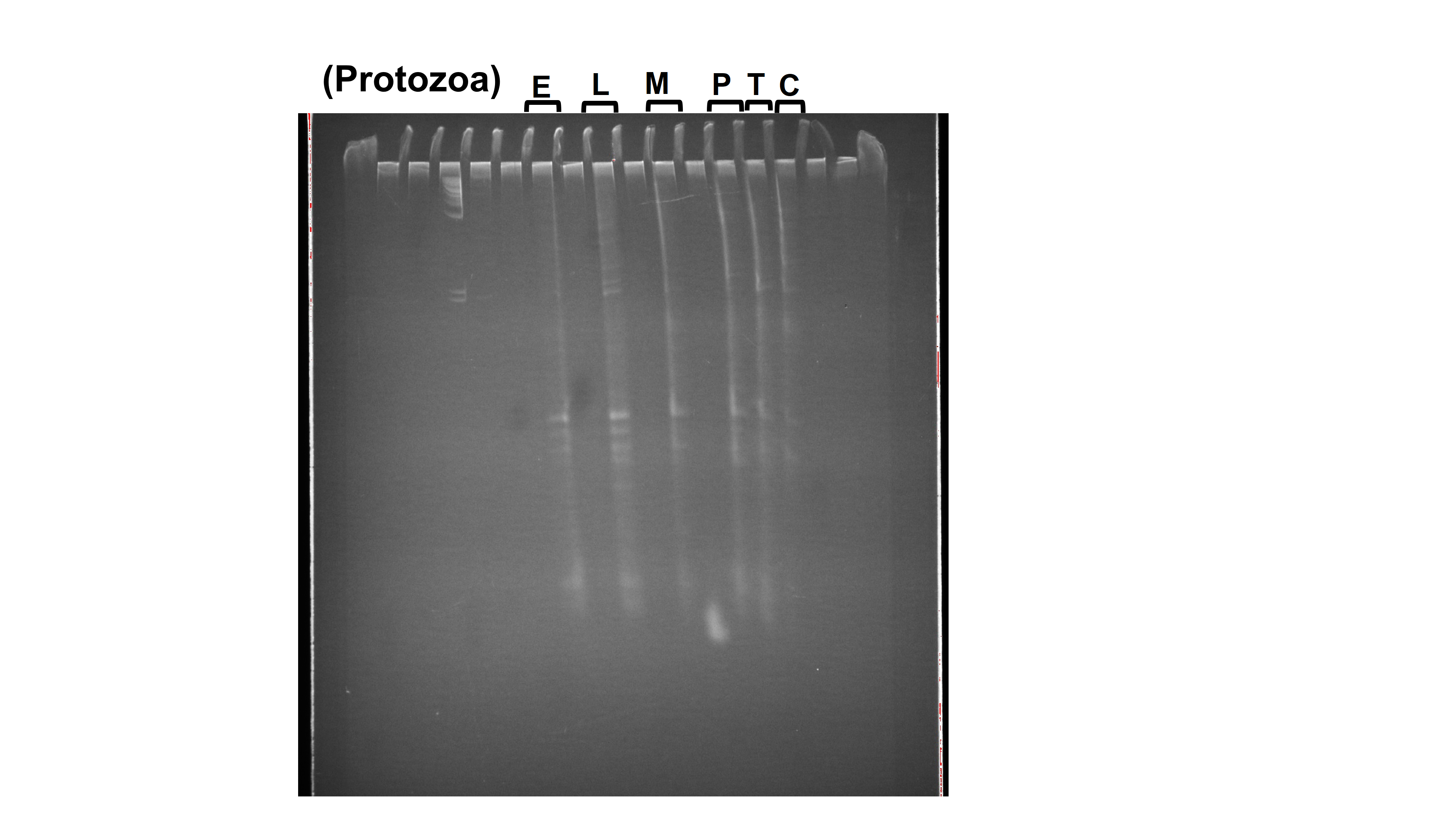


Figure S7. Original DGGE-Gel bands showing population of **protozoa.** The anode biofilm developed in MFCs inoculated with different PWE-treated sludge; Eucalyptus globulus (E), Leucaena leucocephala (L), Mentha piperita (M), Psidium guajava (P), Terminalia chebula (T) and (C) as control, representing mixed anaerobic sludge without PWE treatment. Image is visualized by using Quantity One Software (https://www.bio-rad.com/en-id/product/quantity-one-1-d-analysis software?ID=1de9eb3a-1eb5-4edb-82d2-68b91bf360fb).


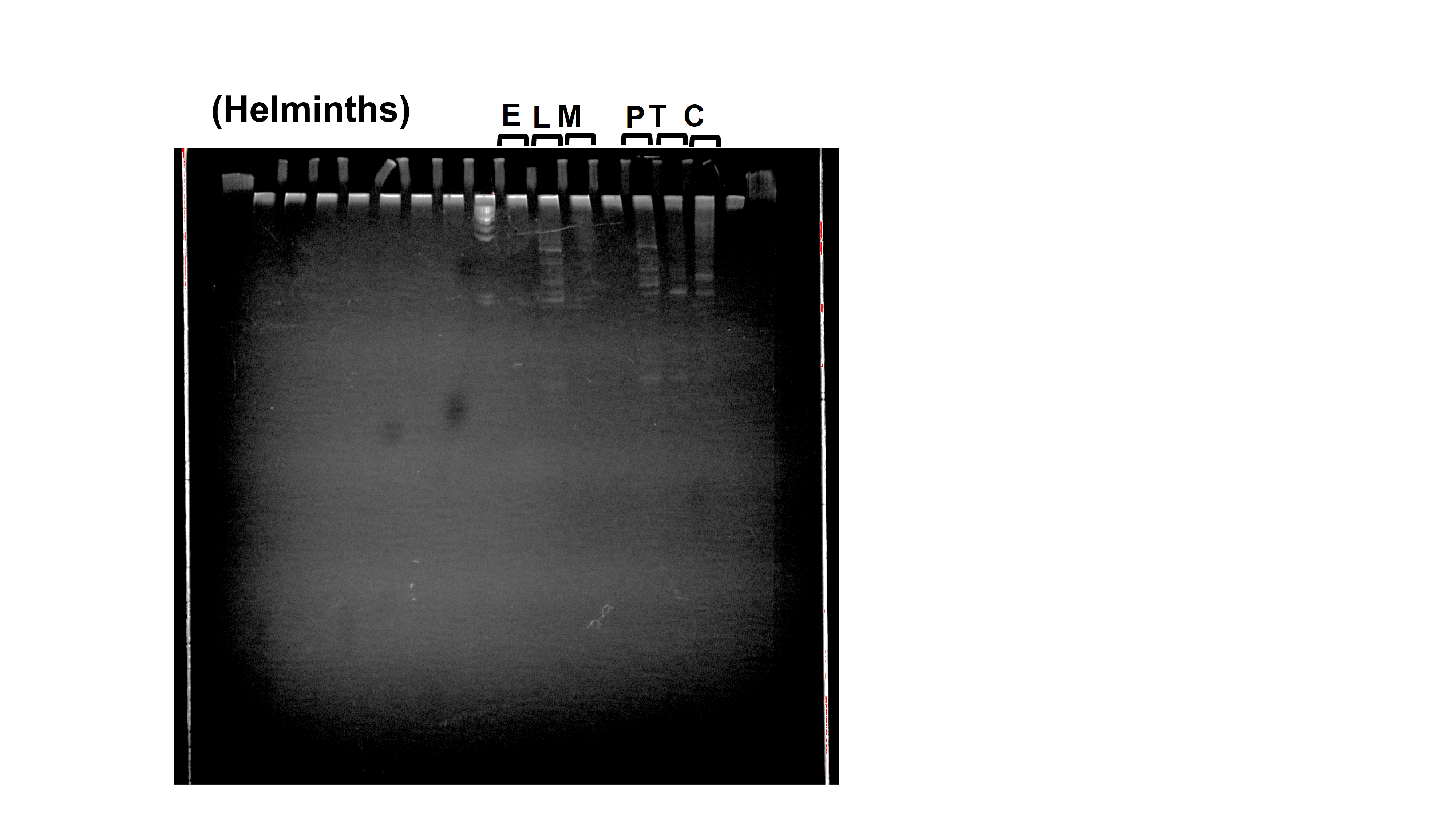


Figure S8. Original DGGE-Gel bands showing population of **helminths.** The anode biofilm developed in MFCs inoculated with different PWE-treated sludge; Eucalyptus globulus (E), Leucaena leucocephala (L), Mentha piperita (M), Psidium guajava (P), Terminalia chebula (T) and (C) as control, representing mixed anaerobic sludge without PWE treatment. Image is visualized by using Quantity One Software (https://www.bio-rad.com/en-id/product/quantity-one-1-d-analysis software?ID=1de9eb3a-1eb5-4edb-82d2-68b91bf360fb).


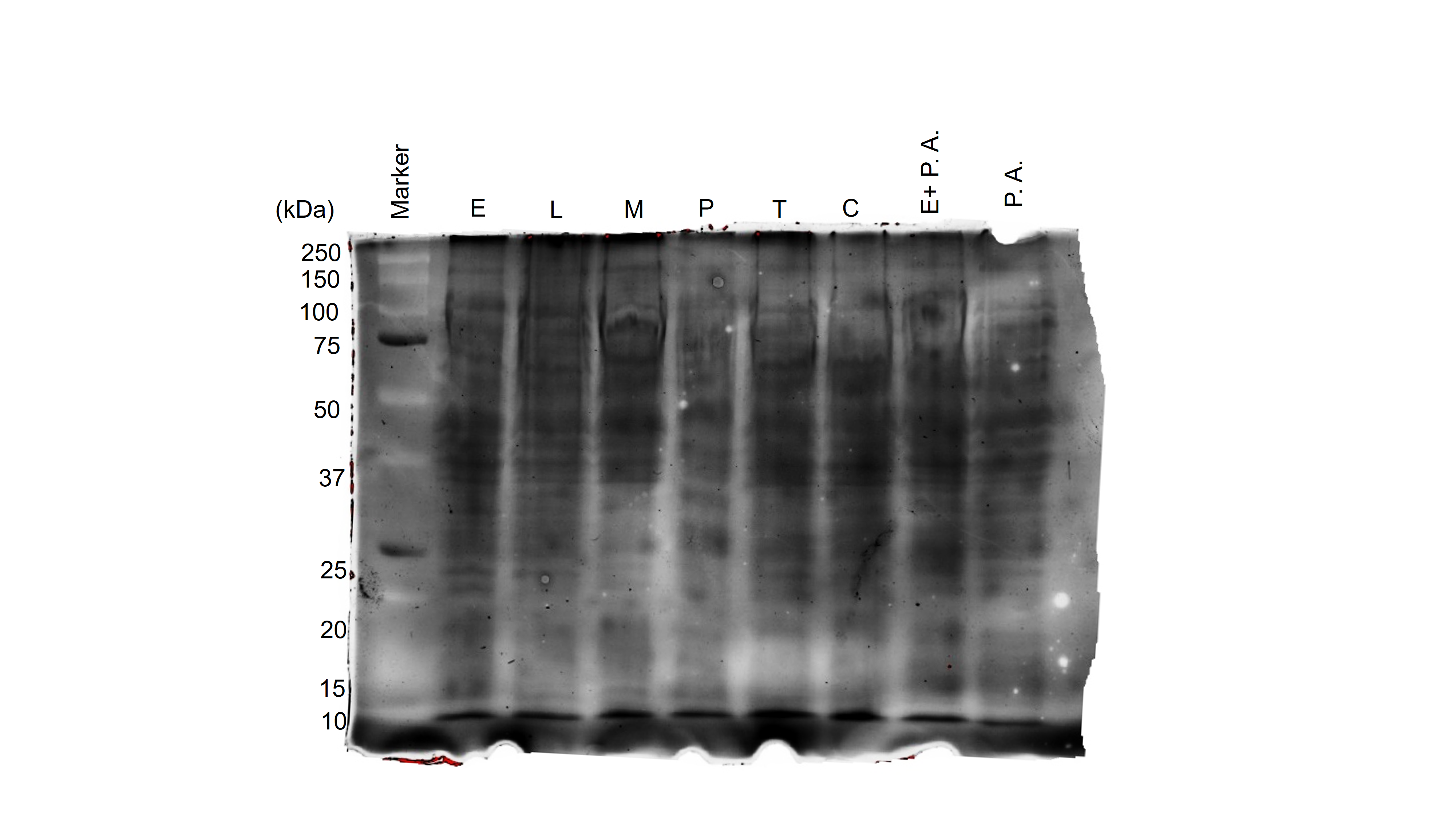


Figure S9. Original Heme-stainng for anode biofilms. Gel bands showing heme containg proteins. The anode biofilm developed in MFCs inoculated with different PWE-treated sludge; *Eucalyptus globulus* (E), *Leucaena leucocephala* (L), *Mentha piperita* (M), *Psidium guajava* (P), *Terminalia chebula* (T) and (C) as control, representing mixed anaerobic sludge without PWE treatment. *Eucalyptus* extracts-treated *P. aeruginosa* (E+ P.A.) and without pretreated *P. aeruginosa* (P.A.). *Image is visualized by using* Quantity One Software (https://www.bio-rad.com/en-id/product/quantity-one-1-d-analysis software?ID=1de9eb3a-1eb5-4edb-82d2-68b91bf360fb)*.*

## Table S1: Quantification of major phytochemicals in plant water extracts

| Plant species | Tannin  (mg TAE.g^-1^) | Total phenols  (mg GAE.g^-1^) | Saponin  (mg.g^-1^) |
| --- | --- | --- | --- |
| *Eucalyptus globulus* | 6.81 ± 0.16 | 163.94 ± 3.6 | 117.93 ± 2.5 |
| *Leucaena leucocephala* | 4.82 ± 0.39 | 144.4 ± 2.9 | 13.2 ± 0.23 |
| *Mentha piperita* | 2.48 ± 0.54 | 49.42 ± 1.2 | 4.24 ± 0.011 |
| *Psidium guajava* | 5.53 ± 0.37 | 62.31 ± 1.24 | 1.8 ± 0.010 |
| *Terminalia chebula* | 2.46 ± 0.02 | 95 ± 1.5 | 3.32 ± 0.013 |

## Table S2: Minimum inhibitory concentration (MIC) of different PWE against mixed bacteria

|  | MIC value | CFU per ml | Reference |
| --- | --- | --- | --- |
| *Eucalyptus globulus (E)* | 3 – 4 mg.ml^-1^ | 5 * 10^8^ | 2 |
| *Leucaena leucocephala (L)* | 3.15 - 25.0 mg.ml^-1^ | 1 * 10^5^ | 3 |
| *Psidium guajava (P)* | 0.5 - 1.0 mg.ml^-1^ | 1 * 10^8^ | 4 |
| *Mentha piperita (M)* | 0.05 - 0.5 mg.ml^-1^ | 1.5 * 10^8^ | 5 |
| *Terminalia chebula (T)* | 1.5 mg.ml^-1^ | 5 * 10^3^ | 6 |

## Table S3: Primers used to amplify the different anodic microbes

| **Microbial Group** | **Primer Sequences** | **Amplicon**  **Size(bp)** |
| --- | --- | --- |
| Electrigens^7^ | Forward 5 ′ - * CCTACGGGAGGCGACAG – 3 ′ | 233 |
|  | Reverse 5 ′ - ATTACCGCGGCTGCTGG – 3 ′ |  |
| Methanogenic  Archea^8^ | Forward 5 ′ - * CCCTACGGGGCGCAGCAG – 3 ′ | 600 |
|  | Reverse 5 ′ - GGATTACARGATTTCAC – 3 ′ |  |
| Protozoa^9^ | Forward 5 ′ - GGTGGTGCATGGCCG – 3 ′ | 200 |
|  | Reverse 5 ′ - * AATTGCAAAGATCTATCCC – 3 ′ |  |
| Helminth^10^ | Forward 5 ′ - * GCAAGTCTGGTGCCAGCAGC – 3 ′ | 630 |
|  | Reverse 5 ′ - CCGTGTTGAGTCAAATTAAG – 3 ′ |  |

*40bp of GC-clamp, *IUPAC code: A (adenine), C (cytosine), G (guanine), T (thymine), R (A or G), Y (C or T), W (A or T), K (G or T), M (A or C), D (A or G or T), H (A or C or T), V (A or C or G), N (A or C or G or T).*

## Table S4. Electrode potential slope analysis of MFC-PAE and MFC-PA

|  | MFC-PAE* | MFC-PA** |
| --- | --- | --- |
| Measured anode potential, mV | -302 | -299 |
| Measured cathode potential, mV | 558 | 553 |
| Measured OCV, mV | 860 | 852 |

*** PAE- *P. aeruginosa* with *Eucalyptus*-extract treatment; ** PA- *P. aeruginosa* without *Eucalyptus*-extract treatment

## Table S5: Deconvolution of Amide (I) region of FTIR spectra for different anodic biofilms

|  | **α-helix (%)** | **β-sheet (%)** | **Turns (%)** | **Random (%)** |
| --- | --- | --- | --- | --- |
| MFC-E | 26.27 | 19.43 | 30.88 | 9.0 |
| MFC-L | - | 100 | - | - |
| MFC-P | 60 | 11.76 | 21.89 | - |
| MFC-M | 39.41 | 6.12 | 27.5 | - |
| MFC-T | 46.95 | 8.19 | 3.57 | 1.56 |
| MFC-C (control) | 24.32 | 5.98 | - | 17.36 |

## Note S1. Preliminary electrochemical evaluation of different PSM and their effect on microbes

Plant water extracts (PWE) of *Eucalyptus globulus* (E), *Leucaena leucocephala* (L),*Psidium guajava* (P), *Mentha piperita* (M), and *Terminalia chebula* (T) were prepared by following the ultrasonic assisted water extraction method^11^, and the presence of different phytochemicals such as tannins, saponins, and total phenolic compounds were quantified as described in methods^12^ (Table S1). The cyclic voltammograms (CVs) of different PWE (without addition of microbes) were assessed, that illustrated both oxidation and reduction peaks (Fig. 1a-1e); which confirmed that sole PSM, present in PWE, could itself act as mediator and participate significantly in electron shuttling^13^. This finding was further corroborated with previous investigation^13^, where it was shown that the functional groups in the PSM [e.g., dihydroxyl (−OH) substituents], if present in the ortho or para position of benzene ring, could significantly exhibit stable reversible electron-shuttling characteristics^14^. The closed-loop area was estimated from the CV curves (V.mA), in the order *Mentha* (4.2) > *Leucaena* (3.9) > *Terminalia* (3.7) > *Eucalyptus* (2.2) > *Psidium* (2.1). However, in case of *Terminalia-*extract, only reduction peaks were detected in CV loop-area; this does not mean lack of electron shuttling property, instead it needs ideal conditions to completely express themselves as described previously^14^.

Previous investigations on the antibacterial effect of PSMs in all five plant water extracts (PWE-PSM) exhibited that the minimum inhibitory concentrations (MICs) ranged from 0.5 mg.ml^-1^ to 5.0 mg.ml^-1^ (Table S2). The MICs were determined only against certain bacterial strains and it was not possible to know a priori, which concentration would be having an effect on the mixed anaerobic consortia enriched in MFCs. Our primary focus was to create external stress on the mixed anaerobic consortia by applying PSMs and investigate their response for EET; hence, a dosing regime was chosen that is likely to induce stress. For example, *Eucalyptus-*PSM dosage with sub-optimal-dose has non-inhibitory effect as reported in earlier investigations^15^. Anaerobic sludge collected from septic tank used as a source of mixed microbes (having volatile suspended solids (VSS) of 25 g.l^-1^, which is equivalent to ~10^6^ CFU cells per ml) was exposed to different plant species-based PWE-PSM dosage of 1 mg of PWE-PSM per ml of sludge in order to assess the effect of inhibitors on mixed consortia.

We carried out the antimicrobial property of each PWE-PSM-dose (1 mg.ml^-1^ anaerobic sludge) using the disc diffusion method^16^ and it was observed that exposure to *Terminalia*-PSM (T) resulted in the greatest zone of inhibition as compared to other PSMs from different plant sp. (Fig. 2a). Varying inhibitory effects of the PSMs at the same dosing concentration could be attributed to the different compositions and characteristics of phytochemicals present in each, for example *Terminalia* extract contains distinct type of hydrolysable tannins (20 - 50%) such as gallic acid, chebulic acid, chebulanin, ellagic acid, terchebulin etc.^17–19^ as compared to the other extracts tested. Whereas, *Eucalyptus* extracts have been shown to be comprised of PSMs such as terpenes, acylphloroglucinols, euglobals, flavonoids, oleuropeic acid, β-triketones^20^.

In order to assess cellular toxicity caused by exposure to the PWE-PSMs, Gram staining and acrylic orange dye-based staining were used to visualize the bacterial cell wall and nucleic acids, respectively. This allowed to distinguish among the cells with intact cell wall, cell wall free cells (spheroplast) and completely/partially lysed cells. Staining was carried out after 24 h of exposure to each PWE-PSM and changes in the cell morphology were visualized by microscopy. It was observed that only in the case of *Eucalyptus* (E) and *Leucaena* (L) PSM treatment, the cells formed chains and cell clusters, which induced in long branched structure formation, that is anomalous growth (Fig. 2b, Gram staining and acrylic orange staining), to combat the toxicity of different polyphenolic content among the extracts (Table S1). This cluster formation was also due to the formation of protein-tannin complexes that affect the biological activity such as increase in chain formation^21^. The chain formation was because of the inhibition of cell division, which resulted in a multinucleated cell with elongation structure hence caused filamentation to adopt the toxicity as previously observed with tannin acid from plant extract and other chemicals like cisplatin and antibiotics^15,22–26^.

The chain formation and spheroplast formation was observed among microbes treated with *Eucalyptus* and *Mentha* PSM-dosing (Fig. 2b). Here, the dispersed layers around the green structures represented as the lysed cellular debris including some nucleic acids released from the neighbouring cells, that generally occurs in microbes under different stresses e.g. antibiotics and starvation^27^. The other PSM-dosing, namely *Psidium* and *Terminalia*, caused only reduction in cell size (Fig. 2b) in comparison with the untreated bacterial cells.

To screen the effects of different PWE-PSMs on EET of the sludge microbes, a high throughput approach i.e. WO_3_-based electrochromic activity test^28^ (where reduced form of WO_3_ develops blue color, which was detected at 920 nm in spectrophotometer) was performed. During EET, a blue color change can be measured optically as a result of reduction in WO_3_^28^. All the PWE-PSMs except *Leucaena-*PSM tested on microbes had a positive effect on the development of blue colour as compared to the untreated microbes, which was refered as the inducible effect of PWE-PSMs on EET. This was due to the difference in the composition of secondary metabolites present in individual plant water extracts. In order to reveal the OD value, which is generated due to the reduction of WO_3_, the OD value of individual plant water extracts before incubation was subtracted from the OD value obtained after incubation. The intensity of OD_920_ was observed to be highest for samples treated with the *Eucalyptus-*PSM (Fig. 2c), suggesting some enhanced effect on EET due to this treatment. We also observed some settleable matters in each vials during WO_3_-based electrochromic test (Fig. 2c, after 24 h incubation) and these are tannin-protein complexes^21^ as observed previously with different PWE-PSMs, where *Eucalyptus-*dose caused a highest filamentatious growth among microbes (Fig. 2b). This is corroborated with previous investigation that, cells with spheroplast (cell growth without cell wall) or partially lazed cell walls condition stretches the periplasm containing the respiratory chain system, including c-type cytochromes, which can efficiently perform EET without any adverse effect^29,30^.

Treatment of microbes with *Leucaena-*PSM exhibited a lower OD value, indicating low EET to WO_3_ reduction; however, the bacterial growth inhibition for *Leucaena-*PSM was not observed during the MIC assay (Fig. 2a) and in addition, *Leucaena-*PSM possess with better electron shuttling properties (Fig. 1b). Hence, the EET is not inhibited, rather it can be assumed that, the electrons must have bypassed towards breaking down of toxic *Leucaena-*PSM e.g. mimosine^31^ (C_8_H_10_N_2_O_4_) rather than WO_3_ reduction (Fig. 2c).

## Note S2. Effect of E-dose on EET mechanism in the anodic biofilm of MFCs

The c-type cytochromes, have compressed globular structures, hence, hydrogen bonds with tannin cannot form strong interaction with these proteins^32^, hence the EET in mixed bacteria was not affected. Hence, the Raman spectroscopic scan of anodic biofilm from each MFC-PWE_microbes_ was performed to confirm the abundance of *c*-type cytochrome. Two distinctive Raman peaks were observed at 1352 and 1592 cm^-1^ in the case of anodic biofilm developed in all MFC-PWE_microbes_ as well as in case of MFC-C (Fig. S2, Raman plot). These two peaks are associated with high-density Cyt-c^33^. Interestingly, in case of the L*-*treated biofilm, four peaks at 572, 918, 1063, and 1132 cm^-1^ were observed, which have been reported to be a consequence of the resonance effect of the porphyrin ring present in the c-type cytochromes^33^. Subsequently, heme-staining with *N*,*N*,*N*,*N*-tetramethylbenzidine staining method was adopted to confirm the presence of heme-containing proteins, which include cytochrome-c in the anodic microbiota (Fig. S2, Heme staining).

The Type IV pili associates with long range electron transfer mechanism in EAB. The Type IV pili is also made up of protein structure, hence the tannin can be bound with Type IV pili and form tannin-protein complexes. Here, a FTIR scan associated with in-vivo detection method was performed to analyze the presence of Type IV pili^34^ on different anodes after 4 months of operation of MFCs. The FTIR scanning in the wavelength range of 1800–700 cm^-1^ was divided into three windows (W1, W2, W3). The abundances of Type IV pili was observed only in W2 (1450-1350 cm^-1^), which is representing the mix region, including protein glycosylation^34^. In the W2 region, Type IV pili was detected and abundance of it was not affected with PWE-treatment (Fig. S2, FTIR plot). Further analysis is required to verify the effect of plant extract on the over expression of c-type cytochromes and Type-IV pilli with specific antibody based western blotting experiment.

The effect of PWE on the metabolic activity in the EAB developed on anode of MFCs was validated through formate dehyrdrogenase (*fdh*) activity test as described previously^60^. The *fdh* activity in anodic biofilm was estimated from the spontaneous generation of NADH in the cell lysate (prepared from the anodic biofilm) by providing sodium formate as the reactant^60^. The anode microbes from MFC-E and MFC-L resulted in increased NADH concentration as compared to the microbes developed in others MFCs (Fig. S2, NADH). The activity of *fdh* acts on NAD^+^/ NADH regeneration and that causes the release of more electrons from EAB, and consecutively leads to increase voltage output in MFC^35^. Hence, it can be concluded that the plant extract treatment did not inhibit the *fdh* activity in anodic biofilms (Fig. S2, *fdh* activity), which indicated that NAD^+^/ NADH regeneration and intracellular redox balance was properly maintained.

The FTIR spectrum within a wavelength range of 1700-1600 cm^-1^ represented amide I region (due to vibration in amide C═O), which is used to determine the protein secondary structures^36^. Thus, the amide-I region obtained from each anodic biofilm developed in different MFCs was deconvoluted into component peaks using secondary analyses and curve-fitting procedure (using Peak fit 7.2 and Origin 8 pro software). The protein secondary structures among microbes in MFC-C were mainly composed of 43.19% of α-helix, 5.98% of β-sheet, 24.32% of Turns and 17.36% of Random structure (Fig. S3 and Table S5); whereas in case of MFC-E, the α-helix and β-sheet conformation were found to be 26.27% and 19.43%, respectively. The decrease in the values of α-helix and increase in β-sheet conformations facilitate high electron flux in the protein structure and also represents a conductive environment^36,37^. Hence the tannin-protein complex had no inhibitory effect on Type IV pili and hence long range electron transfer mechanisms.

## Note S3. COD removal and coulombic efficiency calculation for microbial fuel cell

The performance of the respective MFCs was evaluated with respect to the COD removal and coulombic efficiency (CE, Fig. S4). An average CE of 44.5 ± 4.1 % and COD removal of 73.5 ± 3.2 % was observed in case of MFC-E followed by MFC-L (CE ­of 43.3 ± 2.3 %, COD removal of 77.8 ± 4.5 %). The MFC-M, MFC-P, and MFC-T demonstrated comparable results with COD removal efficiency of 75.3 ± 3.7%, 74.8 ± 3.3%, 70.0 ± 2.4%, respectively, and corresponding CE of 39.6 ± 2.5%, 37.8 ± 2.3%, 34.1 ± 1.8%; whereas, in case of MFC-C, the CE of 27.1 ± 1.1 % was obtained at a COD removal efficiency of 84.1 ± 4.2 %. This average COD removal efficiency of 70 - 75% for MFC inoculated with PSM pre-treated sludge as compared to 84% in control MFC-C reflected that the distinct PSM present in the PWE have modified the microbial metabolic activity properties of the mixed anaerobic inoculum consortium.

# References

1. Ghadge, A. N. & Ghangrekar, M. M. Development of low cost ceramic separator using mineral cation exchanger to enhance performance of microbial fuel cells. *Electrochim. Acta* **166**, 320–328 (2015).

2. Chouhan S, Sharma K, Guleria S. Antimicrobial activity of some essential oils—present status and future perspectives. *Medicines.* **4**, 58 (2017).

3. Abu Zarin, M., Wan, H. Y., Isha, A. & Armania, N. Antioxidant, antimicrobial and cytotoxic potential of condensed tannins from *Leucaena leucocephala* hybrid-Rendang. *Food Sci. Hum. Wellness.* **5,** 65-75 (2016).

4. Sanches, N. R., Cortez, D. A. G., Schiavini, M. S., Nakamura, C. V. & Dias Filho, B. P. An evaluation of antibacterial activities of *Psidium guajava (L.).* *Brazilian Arch. Biol. Technol.* **48**, 429-436 (2005).

5. Alexa, E. *et al.* Phytochemical screening and biological activity of *Mentha × piperita L.* and *Lavandula angustifolia Mill.* extracts. *Anal. Cell. Pathol.* **2018**, 2678924 (2018).

6. Mandeville, A. & Cock, I. E. Terminalia chebula Retz. Fruit extracts inhibit bacterial triggers of some autoimmune diseases and potentiate the activity of Tetracycline. *Indian J. Microbiol.* **2018**, 2678924 (2018).

7. Zhang, Y., Min, B., Huang, L. & Angelidaki, I. Generation of electricity and analysis of microbial communities in wheat straw biomass-powered microbial fuel cells. *Appl. Environ. Microbiol.* **75**, 3389–3395 (2009).

8. Luton, P. E., Wayne, J. M., Sharp, R. J. & Riley, P. W. The mcrA gene as an alternative to 16S rRNA in the phylogenetic analysis of methanogen populations in landfill. *Microbiology* **148**, 3521–3530 (2002).

9. Regensbogenowa, M. *et al.* Assessment of ciliates in the sheep rumen by DGGE. *Lett. Appl. Microbiol.* **39**, 144–147 (2004).

10. Foucher, A. & Wilson, M. Development of a polymerase chain reaction-based denaturing gradient gel electrophoresis technique to study nematode species biodiversity using the 18s rDNA gene. *Mol. Ecol. Notes* **2**, 45–48 (2002).

11. Gharekhani, M., Ghorbani, M. & Rasoulnejad, N. Microwave-assisted extraction of phenolic and flavonoid compounds from *Eucalyptus camaldulensis Dehn* leaves as compared with ultrasound-assisted extraction. *Lat. Am. Appl. Res.* **42**, 305–310 (2012).

12. Parimelazhagan, T. *Pharmacological assays of plant-based natural products*. (ed. Parimelazhagan, T.), Springer. **71,** 188 (2016).

13. Chen, B. Y., Liao, J. H., Hsu, A. W., Tsai, P. W. & Hsueh, C. C. Exploring optimal supplement strategy of medicinal herbs and tea extracts for bioelectricity generation in microbial fuel cells. *Bioresour. Technol.* **256**, 95–101 (2018).

14. Chen, B. Y. *et al.* Deciphering biostimulation strategy of using medicinal herbs and tea extracts for bioelectricity generation in microbial fuel cells. *Energy* **161**, 1042–1054 (2018).

15. Henis, Y., Tagari, H. & Volcani, R. Effect of water extracts of *Carob Pods,* Tannic Acid, and their derivatives on the morphology and growth of microorganisms. *Appl. Microbiol.* **12**, 204–209 (1964).

16. Balouiri, M., Sadiki, M. & Ibnsouda, S. K. Methods for in vitro evaluating antimicrobial activity: A review. *J. Pharm. Anal.* **6**, 71–79 (2016).

17. Kundu, A. P. & Mahato, S. B. Triterpenoids and their glycosides from *Terminalia chebula*. *Phytochemistry* **32**, 999–1002 (1993).

18. Juang, L. J., Sheu, S. J. & Lin, T. C. Determination of hydrolyzable tannins in the fruit of *Terminalia chebula Retz.* by high-performance liquid chromatography and capillary electrophoresis. *J. Sep. Sci.* **27**, 718–724 (2004).

19. Malekzadeh, F., Ehsanifar, H., Shahamat, M., Levin, M. & Colwell, R. R. Antibacterial activity of black myrobalan (*Terminalia chebula Retz*) against *Helicobacter pylori.* *Int. J. Antimicrob. Agents* **18**, 85–88 (2001).

20. Singh, I. P. *Eucalyptus*: a rich source of bioactive secondary metabolites. *Curr. Res. Inf. Pharm. Sci.* **4**, 9–13 (2003).

21. Nelson, K. E., Pell, A. N., Schofield, P. & Zinder, S. Isolation and characterization of an anaerobic ruminal bacterium capable of degrading hydrolyzable tannins. *Appl. Environ. Microbiol.* **61**, 3293–3298 (1995).

22. Patil, S. A., Górecki, K., Hägerhäll, C. & Gorton, L. Cisplatin-induced elongation of *Shewanella oneidensis* MR-1 cells improves microbe-electrode interactions for use in microbial fuel cells. *Energy Environ. Sci.* **6**, 2626–2630 (2013).

23. Paranjape, S. S. & Shashidhar, R. Comparison of starvation induced persister cells with antibiotic induced persister cells. *Curr. Microbiol.* **76**, 1495-1502. (2019).

24. Stenvang, M. *et al.* Epigallocatechin gallate remodels overexpressed functional amyloids in *Pseudomonas aeruginosa* and increases biofilm susceptibility to antibiotic treatment. *J. Biol. Chem.* **291**, 26540-26553 (2016).

25. Cho, Y. S., Schiller, N. L., Kahng, H. Y. & Oh, K. H. Cellular responses and proteomic analysis of *Escherichia coli* exposed to green tea polyphenols. *Curr. Microbiol.* **55**, 501–506 (2007).

26. Cui, Y. *et al.* AFM study of the differential inhibitory effects of the green tea polyphenol (-)-epigallocatechin-3-gallate (EGCG) against Gram-positive and Gram-negative bacteria. *Food Microbiol.* **29**, 80–87 (2012).

27. Ramijan, K. *et al.* Stress-induced formation of cell wall-deficient cells in filamentous actinomycetes. *Nat. Commun.* **9**, (2018).

28. Sharma, I. & Ghangrekar, M. M. Screening anodic inoculums for microbial fuel cells by quantifying bioelectrogenic activity using tungsten trioxide quantum rods. *Bioresour. Technol.* **252**, 66–71 (2018).

29. Smith, L. Structure of the bacterial respiratory-chain system respiration of *Bacillus subtilis* spheroplasts as a function of the osmotic pressure of the medium. *BBA - Biochim. Biophys. Acta* **62**, 145–152 (1962).

30. Spiller, H. Photophosphorylation Capacity of stable spheroplast preparations of anabaena . *Plant Physiol.* **66**, 446–450 (1980).

31. Derakhshani, H., Corley, S. W. & Al Jassim, R. Isolation and characterization of mimosine, 3, 4 DHP and 2, 3 DHP degrading bacteria from a commercial rumen inoculum. *J. Basic Microbiol.* **56**, 580–585 (2016).

32. Hagerman, A. E. Chemistry of tannin-protein complexation. in *Chemistry and Significance of Condensed Tannins.* Hemingway R.W., Karchesy J.J., Branham S.J. (eds). Springer, Boston, MA, 323-333 (1989).

33. Virdis, B., Harnisch, F., Batstone, D. J., Rabaey, K. & Donose, B. C. Non-invasive characterization of electrochemically active microbial biofilms using confocal Raman microscopy. *Energy Environ. Sci.* **5**, 7017 (2012).

34. Bosch, A. *et al.* Type-IV pili spectroscopic markers: Applications in the quantification of piliation levels in Moraxella bovis cells by a FT-IR ANN-based model. *J. Biophotonics* **3**, 522–533 (2010).

35. Han, S., Gao, X., Ying, H. & Zhou, C. C. NADH gene manipulation for advancing bioelectricity in *Clostridium ljungdahlii* microbial fuel cells. *Green Chem.* **18**, 2473–2478 (2016).

36. Zhao, H.-Z., Du, Q., Li, Z.-S. & Yang, Q.-Z. Mechanisms for the direct electron transfer of cytochrome c induced by multi-walled carbon nanotubes. *Sensors (Basel).* **12**, 10450–62 (2012).

37. Reguera, G. Microbes, cables, and an electrical touch. *International Microbiology.* **18**, 151–157 (2015).
